# Low SVEP1 in intrahepatic cholangiocarcinoma mediates phenotype switching-driven metastasis by Jag2/Notch1/Hes5

**DOI:** 10.1038/s41419-025-08170-2

**Published:** 2025-11-28

**Authors:** Lu Chen, Zhiqiang Han, Xiangdong Tian, Kangwei Zhu, Wenchen Gong, Yun Liu, Yimeng Wang, Yuren Xia, Peipei Song, Wei Tang, Norihiro Kokudo, Liwei Chen, Yi Luo, Yuchao He, Tianqiang Song, Hua Guo

**Affiliations:** 1 Tianjin Medical University Cancer Institute and Hospital, National Clinical Research Center for Cancer, Tianjin Key Laboratory of Digestive Cancer, Tianjin’s Clinical Research Center for Cancer, State Key Laboratory of Druggability Evaluation and Systematic Translational Medicine, Tianjin, China; 2 Department of Hepatobiliary Cancer, Liver cancer research center, Tianjin Medical University Cancer Institute and Hospital, Tianjin, China; 3 Department of Hepato-Biliary-Pancreatic Surgery, National Center for Global Health and Medicine, Tokyo, Japan; 4 Department of Tumor Cell Biology, Tianjin Medical University Cancer Institute and Hospital, Tianjin, China; 5 Department of Anesthesiology, Tianjin Medical University Cancer Institute and Hospital, Tianjin, China; 6 Department of Endoscopy, Tianjin Medical University Cancer Institute and Hospital, Tianjin, China; 7 Department of Pathology, Tianjin Medical University Cancer Institute and Hospital, Tianjin, China; 8 Center for Clinical Sciences, National Center for Global Health and Medicine, Tokyo, Japan; 9 International Health Care Center, National Center for Global Health and Medicine, Tokyo, Japan

**Keywords:** Epithelial-mesenchymal transition, Cancer microenvironment

## Abstract

Intrahepatic cholangiocarcinoma (ICC) is a distinct and increasingly prevalent subtype of cholangiocarcinoma arising from the epithelial cells of the intrahepatic bile ducts. Its molecular diversity contributes to its highly aggressive nature and resistance to chemotherapy. SVEP1 (sushi, Von Willebrand factor type A, EGF, and pentaxin) is a multi-domain extracellular matrix (ECM) protein that is vital for embryogenesis, cell-cell adhesion, and the maintenance of epidermal differentiation. However, the specific effect of SVEP1 on the occurrence and progression of ICC remains poorly understood. Therefore, this study aims to examine the role of SVEP1 in ICC. We first identified SVEP1 using high-throughput RNA sequencing in two groups of patients with ICC with different disease-free survival rates. We further analyzed the expression pattern of SVEP1 in ICC using various public datasets and clinical tissue samples, exploring the correlation between SVEP1 depletion and ICC clinical prognosis. The regulatory role of SVEP1 depletion in ICC progression was studied using in vitro and in vivo experiments. We found that decreased SVEP1 expression positively correlates with early recurrence and shorter overall survival in ICC. Moreover, SVEP1 downregulation was correlated with multiple poor prognostic parameters, including positive lymph nodes, satellite nodes, and high Ki-67 expression. Downregulated SVEP1 expression promoted ICC cell proliferation, chemotactic migration, and invasion in vitro, as well as tumor growth and lung metastasis in vivo. These effects were mediated by EMT phenotype switching through the activation of the Jag2/Notch1/Hes5 pathway. Our findings enhance the understanding of the novel mechanisms driving ICC progression and metastasis, suggesting that SVEP1 is a potential biomarker for ICC diagnosis.

## Introduction

Cholangiocarcinomas (CCA) rank as the sixth most lethal cancer globally, accounting for approximately 2% of all cancer-related deaths annually [1]. Its incidence has markedly risen in recent years, with only 30–40% of patients eligible for curative surgery owing to the frequent distant metastases at initial diagnosis [2]. ICC arises from the malignant transformation of biliary epithelial cells within the hepatic parenchyma and accounts for approximately 10–15% of all CCA [3]. While radical resection remains the primary treatment of hepato-biliary cancer, local recurrence, distant metastasis, and the lack of effective, comprehensive therapies contribute to poor long-term outcomes [4, 5]. Nearly half of the patients with ICC experience recurrence within a year post-surgery, with a dismal 5-year overall survival rate [4]. Therefore, a comprehensive understanding of ICC metastasis is crucial for guiding future therapeutic strategies and alleviating the disease burden.

Metastasis is a fundamental biological characteristic of malignant tumors and remains the leading cause of death among patients with cancer in clinical practice [6, 7]. Substantial evidence underscores the pivotal role of phenotypic switching in tumor cell behavior, influencing tumor growth and metastasis [8]. Previous studies indicate that primary liver cancers exhibiting phenotypic switching demonstrate enhanced infiltration and metastatic potential, correlating with shorter disease-free survival (DFS) and overall survival (OS) [9, 10]. Cellular phenotypic reprogramming primarily involves three categories: dedifferentiation, transdifferentiation, and termination of differentiation [11]. Among these, epithelial-mesenchymal transition (EMT) is a well-known process characterized by reduced cell-cell adhesion, loss of apical-basal polarity, increased cell motility and invasiveness, and the acquisition of mesenchymal properties [12, 13]. Despite their significance in cancer progression and therapeutic resistance, the precise mechanisms controlling cancer cell phenotype switching remain poorly understood. Therefore, further exploration is necessary to identify the key molecules involved in EMT-like phenotype-switching processes.

ECM proteins are crucial in mediating interactions between cells and the ECM. Alterations in the genes encoding ECM proteins drive cellular phenotype switching during tumor occurrence and development [14]. SVEP1 is a multi-domain ECM protein that was discovered in bone, periosteum, and bone marrow tissues in 2006 [15]. Recent research has highlighted its role in embryonic development, cell adhesion, differentiation, and maintenance of tissue microenvironment homeostasis [16, 17]. Previous studies demonstrate that decreased SVEP1 expression in hepatocellular carcinoma (HCC) induces malignant phenotype transformation, promoting multiple metastases and invasion into osteophagy in vivo [18]. Furthermore, recent findings from our group indicate that the SVEP1^low^ subclone in HCC correlates significantly with high tumor heterogeneity—a cancer stem cell-like phenotype, and poor prognosis [19, 20]. Therefore, understanding the roles of SVEP1 and its interactors in cancer is crucial for leveraging its prognostic and therapeutic potential.

To date, integrin α9β1 is the only protein known to directly interact with SVEP1 [21]. Studies show that SVEP1 facilitates cell-cell adhesion in an integrin α9β1-dependent manner, with its affinity for integrin α9β1 surpassing that of other known ligands [22]. Among the 24 reported integrin heterodimers, integrin α9β1 remains one of the least studied types. Recent research reveals its role in tumor progression, highlighting its involvement in multiple biological processes associated with tumor initiation and metastasis via interactions with its ligands [23]. However, the exact role of aberrant interactions between SVEP1 and integrin α9β1 in cancer development remains poorly understood, necessitating further elucidation.

Therefore, this study aims to examine the role of SVEP1 in ICC. Through RNA sequencing, the differential expression of SVEP1 was observed in two groups of patients with ICC with varying DFS. Validation using TCGA and GEO databases, along with IHC staining of a 113-case ICC tissue array, confirmed that reduced SVEP1 expression in ICC correlates significantly with early postoperative recurrence and poor prognosis. We also discovered that the interaction between SVEP1 and integrin α9β1 regulates phenotype switching in ICC cells. The molecular mechanism underlying this regulation was explored, revealing that reduced binding between SVEP1 and integrin α9β1 activates the Jag2/Notch1/Hes5 signaling axis. Consequently, this activation promotes EMT, enhancing the metastatic potential. These findings deepen our understanding of cellular phenotypic switching in ICC progression, highlighting SVEP1 as a potential biomarker and therapeutic target in patients with metastatic ICC.

## Results

### SVEP1 identification and its predicting value for clinical prognosis in ICC

**Fig. 1:**
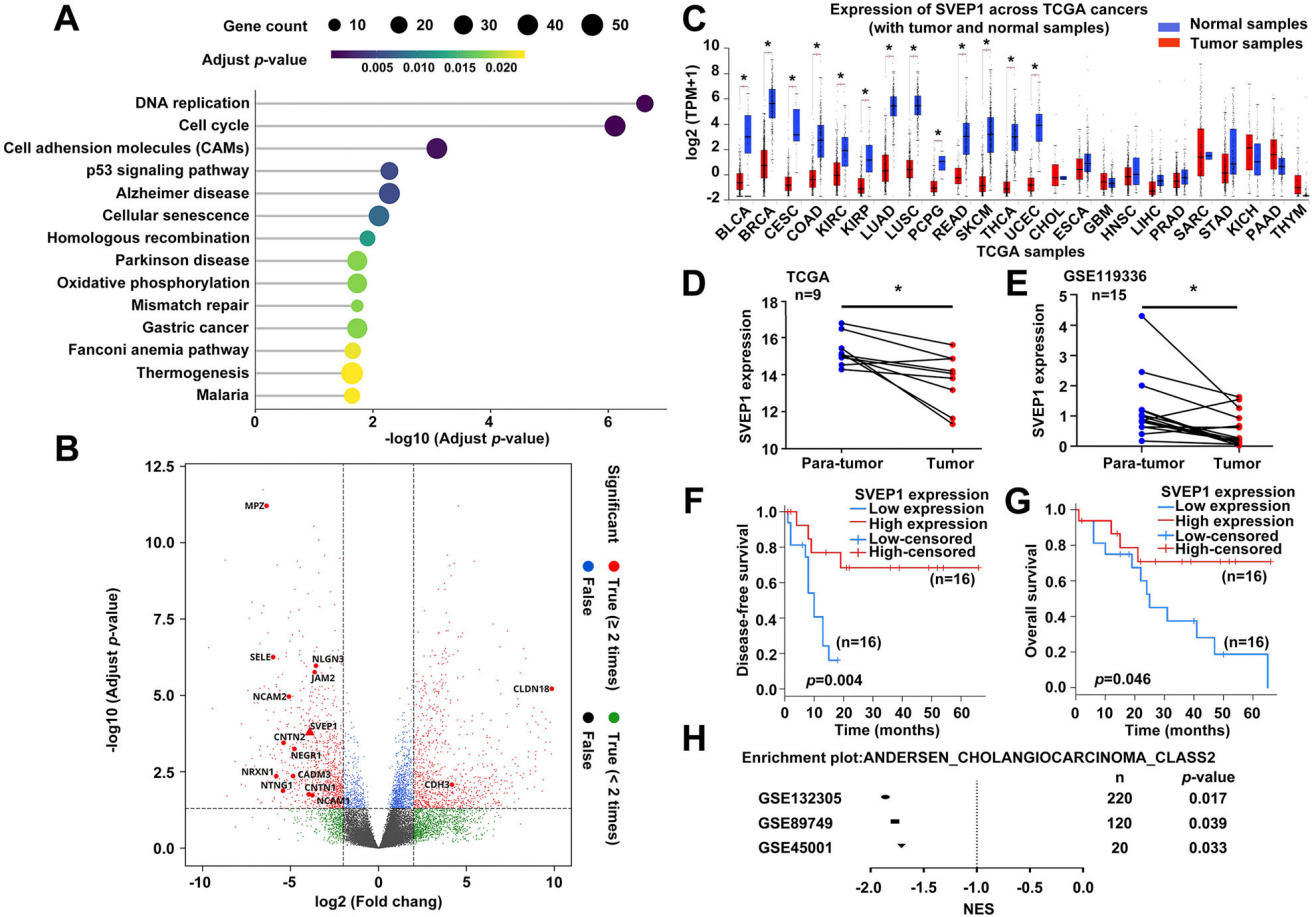
SVEP1 depletion in ICC tissues correlates with poor prognosis. **A** Pathway enrichment analysis based on DEGs between high-recurrence group (7 cases) and low-recurrence (10 cases) groups in patients with ICC using high-throughput RNA sequencing. **B** Identification of specific DEGs in CAMs pathway. **C** The mRNA levels of SVEP1 in tumor and normal tissues in 24 histotypes of pan-cancer analyzed via the TCGA database. Validation of SVEP1 expression in CCA tumor tissues compared to para-tumor tissues using TCGA (**D**) and GSE119336 (**E**) datasets. **F**, **G** Kaplan–Meier survival analysis using TCGA datasets to explore the correlation between SVEP1 expression and DFS and OS of patients with CCA (**H**). Analysis of three GEO datasets (GSE132305, GSE89749, GSE45001) to further confirm the correlation between SVEP1 expression and clinical prognosis. *p < 0.05.

To investigate the mechanisms underlying recurrence and metastasis in ICC, we conducted high-throughput RNA sequencing on two groups of patients with ICC with similar TNM stages according to the American Joint Committee on Cancer (AJCC) staging manual 8th edition while observing differences in DFS (Supplementary Table 2). The high-recurrence group, defined by DFS < 12 months (7 cases), contrasted with the low-recurrence group, consisting of patients with a DFS > 24 months (10 cases). Figure 1A depicts the pathway enrichment analysis of differentially expressed genes (DEGs) in these groups, highlighting DNA replication, cell cycle, and cell adhesion molecules (CAMs) as the top three significantly enriched pathways. Studies reveal that CAM degradation is crucial for reducing tumor cell adhesion and promoting detachment from primary sites, leading to metastatic formation [24]. However, specific CAMs pivotal in ICC metastasis remain unknown. To address this gap, DEGs within CAMs were analyzed using thresholds of *p* ≤ 0.05 and | log2 FC| ≥ 1 (Fig. 1B). SVEP1, a gene of significant interest in our previous studies on hepatocellular carcinoma (HCC) [18–20], was identified among the downregulated mRNAs in the high recurrence group (Fig. 1B). However, the expression patterns and functional roles of SVEP1 in other cancer histotypes, including ICC, remain unclear.

To address this knowledge gap, we analyzed SVEP1 mRNA levels across 24 tumor histotypes using TCGA database. Our findings revealed that the SVEP1 levels were reduced in 18 of the 24 cancer types (Fig. 1C). SVEP1 downregulation in CCA tumor tissues was significantly more pronounced than that in the corresponding para-tumor tissues in TCGA (paired, *n* = 9) and GSE119336 (paired, *n* = 15) databases (Fig. 1D, E). Survival analysis using TCGA datasets revealed a significant positive correlation between high SVEP1 expression in tumors and extended DFS (Fig. 1F, *p* = 0.004) and OS (Fig. 1G, *p* = 0.046) in patients with CCA. Additionally, Gene set enrichment analysis (GSEA) confirmed that low SVEP1 expression was associated with poorer clinical prognosis of patients with CCA based on mRNA data from three other GEO datasets: GSE132305 (*n* = 220, *p* = 0.046), GSE89749 (*n* = 120, *p* = 0.039), and GSE45001 (*n* = 20, *p* = 0.033) (Fig. 1H and Supplementary Fig. 1A–C). While the molecular mechanisms of SVEP1 in regulating the early recurrence and poor prognosis of CCA, particularly ICC, remain incompletely understood, our study highlights SVEP1 as a novel and promising prognostic factor in ICC.

### SVEP1 expression pattern and its correlation with clinicopathological characteristics of ICC

**Fig. 2:**
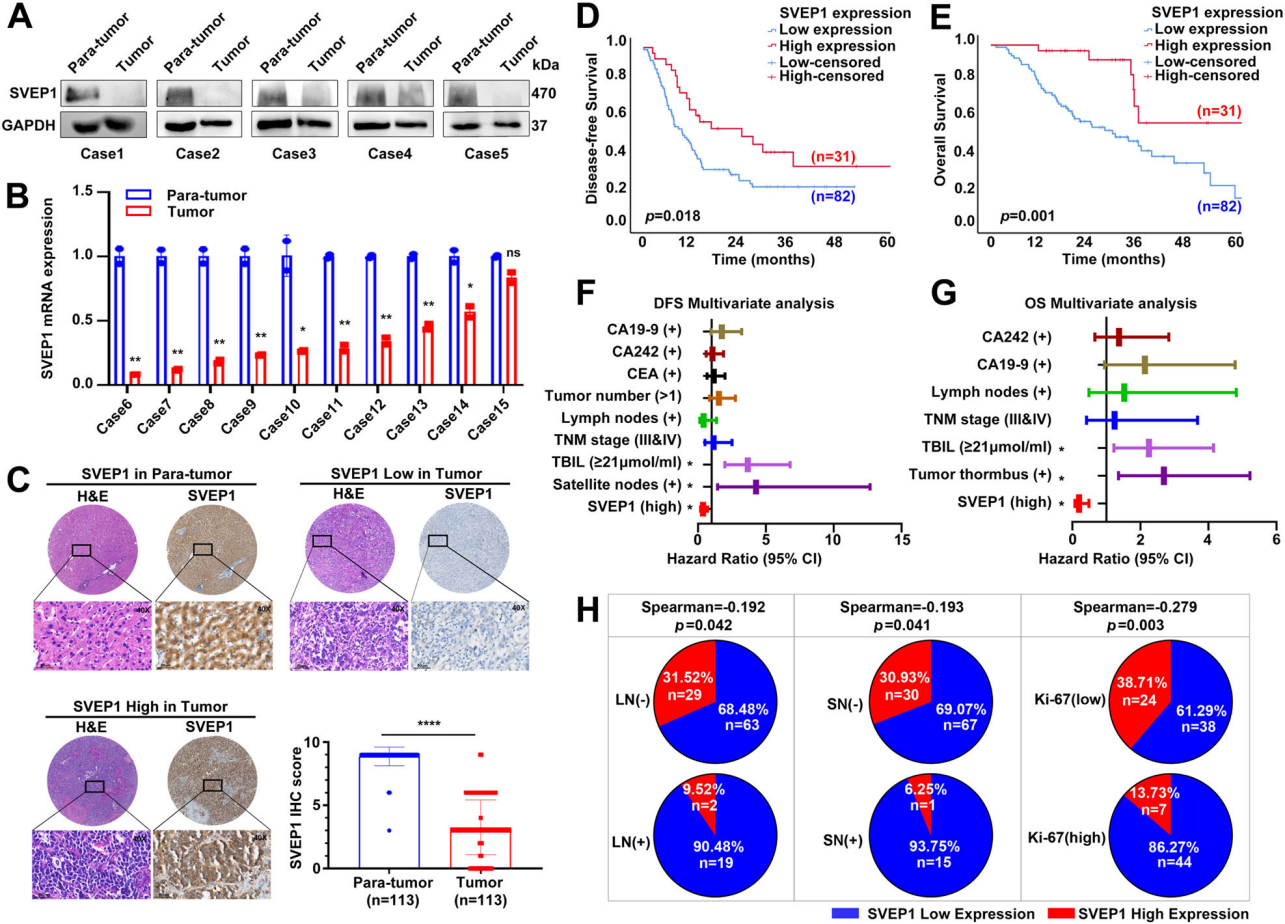
SVEP1 depletion is an independent prognostic risk factor in ICC. WB (**A**) and RT-PCR (**B**) analysis comparing SVEP1 expression levels between ICC tumor tissues and its corresponding para-tumor tissues (5 pairs and 10 pairs, respectively). **C** Representative IHC staining images showing examples of para-tumor tissues, high-SVEP1 expression tumors (score ≥ 6), and low-SVEP1 expression tumors (score < 6) in 113 ICC cases (Scale bars, 50 μm). Kaplan–Meier plots comparing 5-year DFS (**D**, *p* = 0.018) and OS (**E**, *p* = 0.001) rates between patients with the high-SVEP1 vs. low-SVEP1 expression. Identification of independent risk factors for DFS (**F**) and OS (**G**) in the cohort of 113 ICC cases according to multivariate analysis. **H** Correlation analysis between SVEP1 IHC scores and clinicopathological parameters, denoted as LN (short for lymph nodes), SN (short for satellite nodules), and Ki-67 expression. ns non-significant, *p < 0.05, **p < 0.01, ****p < 0.0001.

To validate the expression pattern of SVEP1 in ICC, we selected three additional ICC cohorts from our center and analyzed SVEP1 expression and localization in paired tumors and corresponding para-tumor tissues using RT-PCR and IHC staining. First, WB analysis of 5 paired freshly frozen ICC samples confirmed that SVEP1 expression was significantly reduced in ICC tumor tissues (Fig. 2A). Consistent with the WB findings, RT-PCR analysis of another 10 pairs of ICC samples showed comparable results (Fig. 2B). Furthermore, IHC staining of a tissue microarray comprising 113 cases revealed a significant decrease in SVEP1 expression in most ICC tumor tissues (*p* < 0.0001), with positive SVEP1 expression primarily observed in the cytoplasm and ECM (Fig. 2C). Subsequently, we analyzed the correlation between SVEP1 expression and clinical prognosis using Kaplan–Meier curves based on SVEP1 IHC staining. Consistent with findings from previous studies, patients with low-SVEP1 staining (*n* = 82) exhibited a significantly poorer DFS (Fig. 2D, *p* = 0.018) and OS (Fig. 2E, *p* < 0.001) than those with high SVEP1 staining (*n* = 31). The average DFS and OS of patients with ICC with low-SVEP1 expression were 18.82 months (18.82 ± 2.16) and 36.98 months (36.98 ± 3.76), respectively, which were significantly shorter than those of patients with high SVEP1 expression (35.44 months, 35.44 ± 5.93, and 65.42 months, 65.42 ± 8.11).

Given the decreased SVEP1 expression in most ICC tumor tissues and its association with poor prognosis, we examined the correlation between SVEP1 expression and other known ICC risk factors associated with short DFS or OS, such as tumor size, lymph node (LN) metastasis, and serum CA19-9 levels [25–27]. Multivariate analysis revealed total bilirubin (TBIL) ≥ 21 μmol/L (*p* < 0.001), positive satellite nodes (SN, *p* = 0.009), and low-SVEP1 expression (*p* = 0.002) as independent risk factors for DFS in our cohort of 113 ICC cases (Fig. 2F and Supplementary Table 3). Additionally, TBIL ≥ 21 μmol/L (*p* = 0.009), positive tumor thrombus (*p* = 0.005), and low-SVEP1 expression (*p* < 0.001) were considered independent risk factors for OS (Fig. 2G and Supplementary Table 3). While TBIL, SN, and tumor thrombus align with previous literature [28–30], our study is the first to identify low-SVEP1 expression as an independent risk factor for DFS and OS in ICC.

We then examined the correlation between low-SVEP1 expression and other risk factors in ICC. SN and LN metastasis, along with Ki-67 expression, were found to be closely correlated with SVEP1 expression levels (Supplementary Table 4). Specifically, 19 of 21 (90.48%) patients with positive LN metastasis exhibited low-SVEP1 expression, compared with 63 of 92 (68.48%) patients in the negative LN metastasis group (Fig. 2H, *p* = 0.042). Furthermore, the incidence of low-SVEP1 expression was significantly higher in the positive SN group than that in the negative SN group (Fig. 2H, 93.75% vs. 69.07%, *p* = 0.041). These differences were particularly more significant between the Ki-67 high and low expression groups (Fig. 2H, 86.27% vs. 61.29%, *p* = 0.003). Additionally, even within the low-risk subgroups for recurrence, such as those with negative LN metastasis (Supplementary Fig. 1D, E), negative SN (Supplementary Fig. 1F, G), and negative Ki-67 (Supplementary Figs. 1H, I, and 2A), decreased SVEP1 expression was identified as a poor prognostic biomarker for DFS and OS. These findings collectively suggest that decreased SVEP1 expression promotes metastasis and proliferation in ICC.

### Downregulation of SVEP1 in ICC increased cell proliferation, migration, and invasion in vitro

**Fig. 3:**
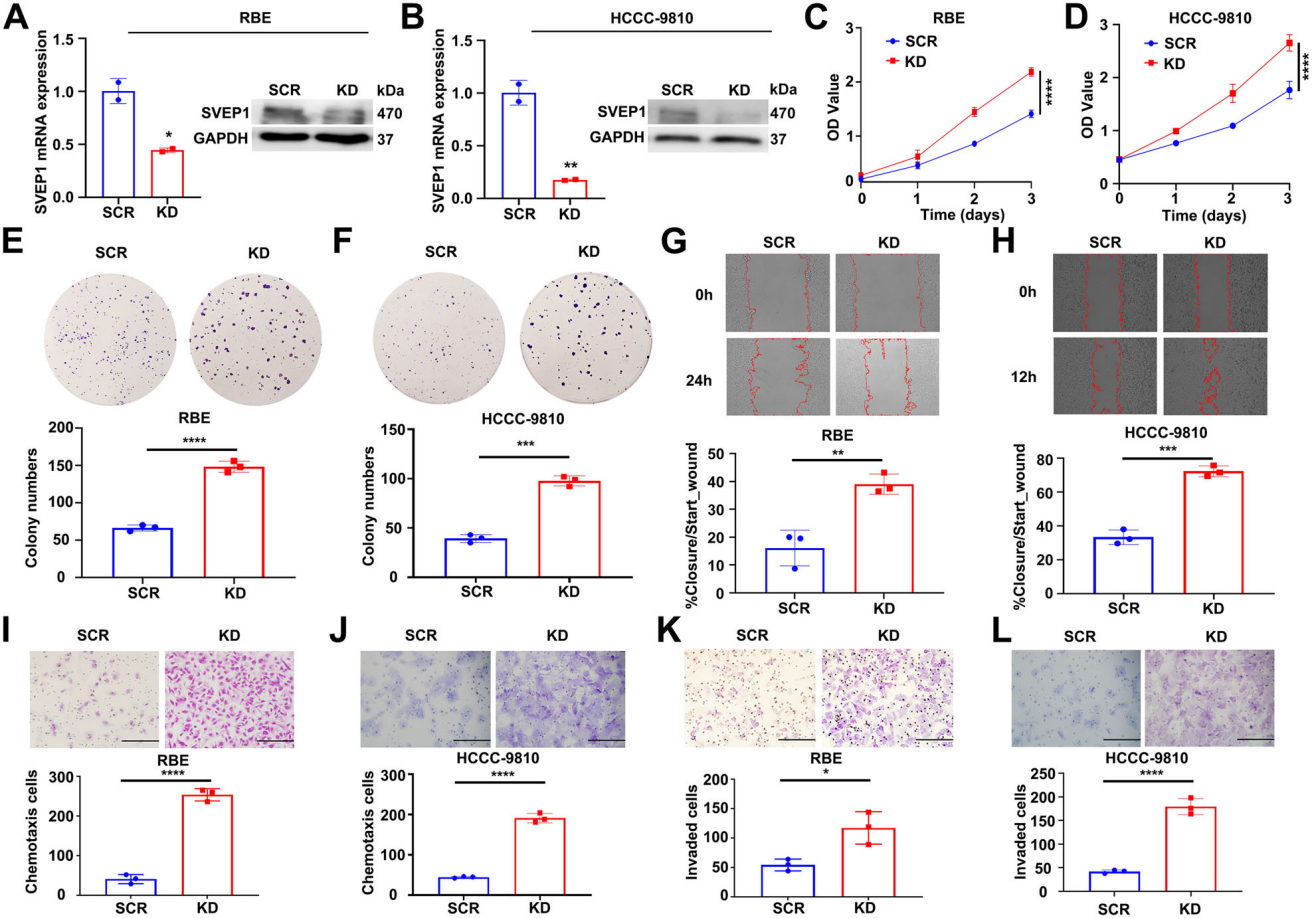
Down-regulation of SVEP1 increases cell proliferation, migration, and invasion in ICC in vitro. WB and RT-PCR analyses showing the SVEP1 knocked down clone obtained in RBE (**A**) and HCCC-9810 (**B**) cell lines, denoted as KD (short for SVEP1 KD). GAPDH was used as a loading control. CCK-8 assay exhibiting differences in proliferation ability between SCR and KD groups in RBE (**C**) and HCCC-9810 (**D**) cell lines. Representative images of 2D colony formation assays in RBE (**E**) and HCCC-9810 (**F**) cells transfected with SVEP1 or control shRNAs. Representative images and quantification of wound closure in RBE (**G**, time = 24 h) and 9810 (**H**, time = 12 h) cells transfected with SVEP1 or control shRNAs after scratching. Representative images of chemotactic migration (transwell assay) in RBE (**I**) and 9810 (**J**) cells transfected with SVEP1 or control shRNAs (Scale bars, 100 μm). Cell invasion assay results comparing SCR and KD groups in RBE (**K**) and HCCC-9810 (**L**) cells using transwell filter chambers. Representative images show invaded cells were calculated (Scale bars, 100 μm). *p < 0.05, **p < 0.01, ***p < 0.001, ****p < 0.0001.

Given the decreased expression of SVEP1, particularly in ICC patients with LN metastasis and Ki-67 positive expression, we investigated the effect of SVEP1 on the proliferation, migration, and invasion of ICC cells. We initially established stable knockdown cell lines using RBE and HCCC-9810 cells and confirmed the efficiency of SVEP1 deletion using WB and RT-PCR (Fig. 3A, B and Supplementary Fig. 2A). Using two different proliferation assays, CCK-8 (Fig. 3C, D and Supplementary Fig. 2B) and plate colony formation (Fig. 3E, F and Supplementary Fig. 2C), we observed that suppressing SVEP1 expression significantly enhanced the proliferative ability of RBE and HCCC-9810 cells compared to those of the respective control groups. This finding aligns with that of previous studies, as SVEP1 expression was strongly negatively correlated with Ki-67 expression, and the proportion of low-SVEP1 expression was significantly higher in ICC patients with Ki-67 positive expression (Fig. 2H and Supplementary Table 4).

We found that downregulating SVEP1 in RBE and HCCC-9810 cells significantly enhanced cell motility, as shown by the results of the wound-healing assay (Fig. 3G, H and Supplementary Fig. 2D). Furthermore, SVEP1 depletion increased the chemotactic ability of ICC cells (Figs. 3I, J and Supplementary Fig. 2E). In the cell invasion experiment (Figs. 3K, L and Supplementary Fig. 2F), significantly fewer SVEP1-depleted RBE and HCCC-9810 cells were invaded compared to control cells. DEGs between RBE/KD and RBE/SCR cells were identified, and subsequent gene ontology (GO) and pathway analyses were conducted, revealing the involvement of DEGs in cell motility, migration, and chemotaxis, thereby indirectly confirming the results of the cellular functional experiments (Supplementary Fig. 3A and Supplementary Table 5). Overall, these in vitro experiments further substantiate the role of SVEP1 depletion in regulating the malignant progression of ICC by promoting proliferation and metastasis, which is consistent with previous clinical findings (Fig. 2 and Supplementary Fig. 3B).

### SVEP1 depletion induced EMT-phenotype switching in ICC

**Fig. 4:**
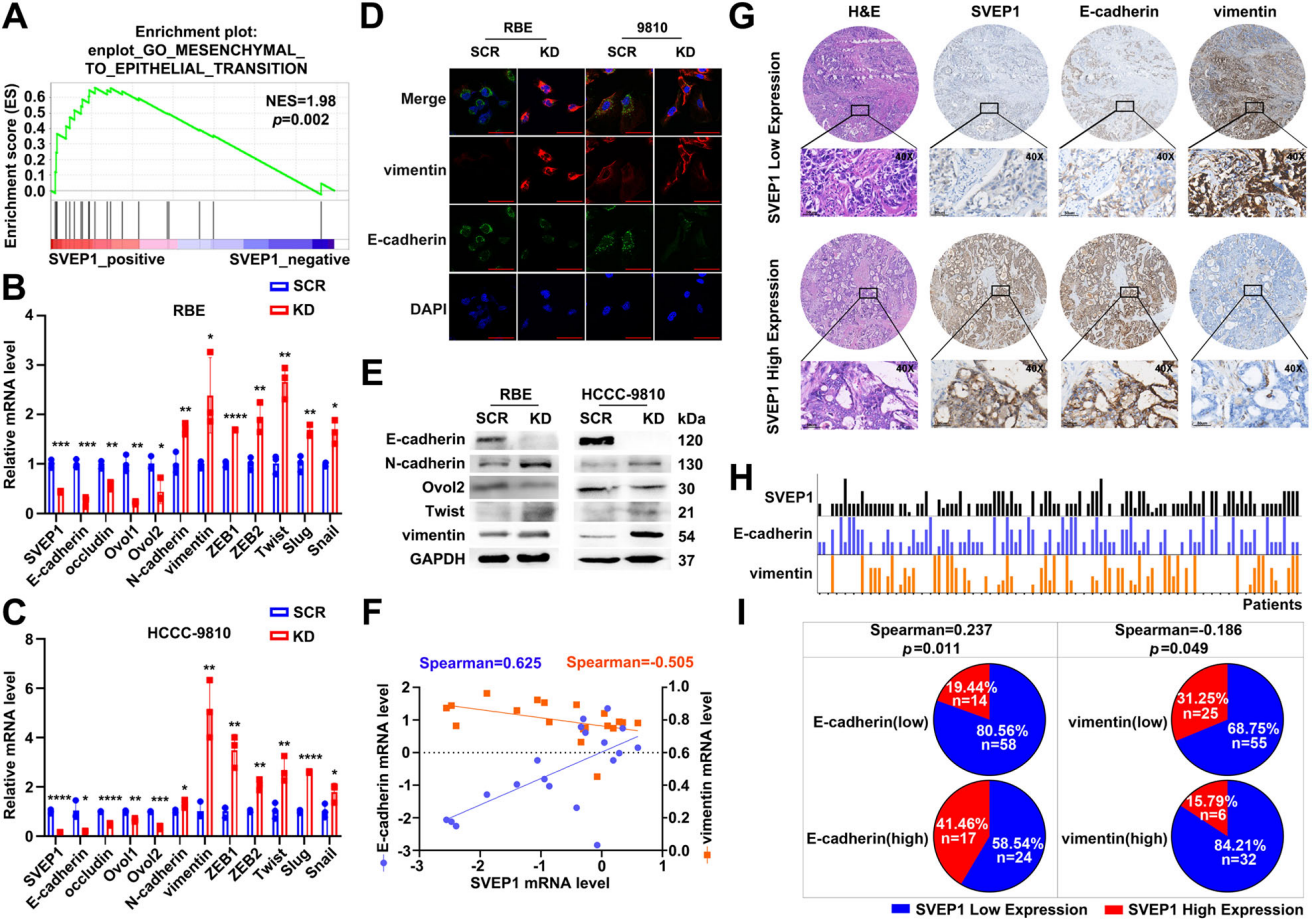
SVEP1 depletion induces EMT-phenotype switching in ICC. **A** GSEA (GSE89749) shows a positive correlation of MET-associated genes with SVEP1 expression. Relative mRNA levels of SVEP1 and EMT-related markers (biomarkers and transcription factors) analyzed via RT-PCR in RBE (**B**) and HCCC-9810 (**C**) cells between SCR and KD groups. **D** Representative immunofluorescence images of RBE and HCCC-9810 cells stained with DAPI (blue) and labeled for E-cadherin (green) and vimentin (red) after transfected with SVEP1 or control shRNAs (Scale bars, 50 μm). **E** WB analysis of SVEP1 and EMT-related markers protein levels in RBE and HCCC-9810 cells transfected with SVEP1 or control shRNAs. **F** RT-PCR analysis showing the correlation between SVEP1 mRNA level and epithelial-marker (blue line, E-cadherin) or mesenchymal-marker (orange line, vimentin) expression, respectively. **G** H&E and IHC staining of 113 cases of patients with ICC (Scale bars, 50 μm). **H** The corresponding relationship between SVEP1 expression and EMT-biomarker expression (black-line, SVEP1; blue-line, E-cadherin; orange-line, vimentin) in each patient shown in detail in (**H**). **I** Correlation analysis between SVEP1 IHC score and E-cadherin or vimentin expression in 113 cases of patients with ICC. ns = non-significant, *p < 0.05, **p < 0.01, ***p < 0.001.

To further identify the molecular pathways linked to SVEP1-regulated genes, we conducted GSEA using mRNA data from GSE89749, revealing significant enrichment of SVEP1-regulated genes associated with mesenchymal-epithelial transition (MET) (Fig. 4A, *p* = 0.002). This finding was further corroborated by analyses of the GSE107102 and GSE89749 datasets (Supplementary Fig. 3C, D). Consistent with the observed increase in proliferation, chemotaxis, and invasive capabilities (Fig. 3), we observed the downregulation of SVEP1 in RBE and HCCC-9810 cells, which markedly induced cellular EMT phenotype switching, as evidenced by the RT-PCR assays. For instance, epithelial-associated genes, such as E-cadherin, occludin, and Ovol1/2, were downregulated following the depletion of SVEP1 in RBE and HCCC-9810 cells. Conversely, the levels of the mesenchymal-associated genes vimentin, N-cadherin, ZEB1/2, Twist, slug, and Snail increased (Fig. 4C, D). Immunofluorescence results further validated the significant downregulation of E-cadherin and upregulation of vimentin in RBE and HCCC-9810 cells after SVEP1 knockdown compared with those of their respective control cells (Fig. 4D). Additionally, the WB assay confirmed the EMT phenotype switching in ICC cells in response to SVEP1 depletion (Fig. 4E).

To evaluate the clinical role of SVEP1 in ICC phenotype switching, we analyzed 19 paired freshly frozen tissues and 113 paired paraffin-embedded ICC tissues to investigate the correlation between SVEP1 and EMT-associated markers. Figure 4F depicts that the SVEP1 mRNA levels exhibited a positive correlation with E-cadherin and a negative correlation with vimentin, indicating a close correlation between reduced SVEP1 expression and EMT phenotype switching in ICC. Through IHC staining and clinical analysis of another large cohort of ICC cases, we further clarified that decreased SVEP1 expression significantly correlated with the loss of the epithelial phenotype and acquisition of the mesenchymal phenotype in ICC (Fig. 4G, H and Supplementary Table 4). Specifically, 58 of 72 (80.56%) patients with low E-cadherin expression exhibited SVEP1 depletion, compared to 24 of 41 (58.54%) patients with high E-cadherin expression (Fig. 4I, *p* = 0.011). Furthermore, the prevalence of patients with SVEP1 depletion differed significantly between groups with varying levels of vimentin expression (Fig. 4I, 55/80, 68.75% vs. 32/38, 84.21%, *p* = 0.049). Therefore, decreased SVEP1 expression may be a key factor in the process of EMT phenotype switching in ICC.

### SVEP1 is critical for maintaining the epithelial phenotype of ICC and inhibiting tumor progression and metastasis in vivo

**Fig. 5:**
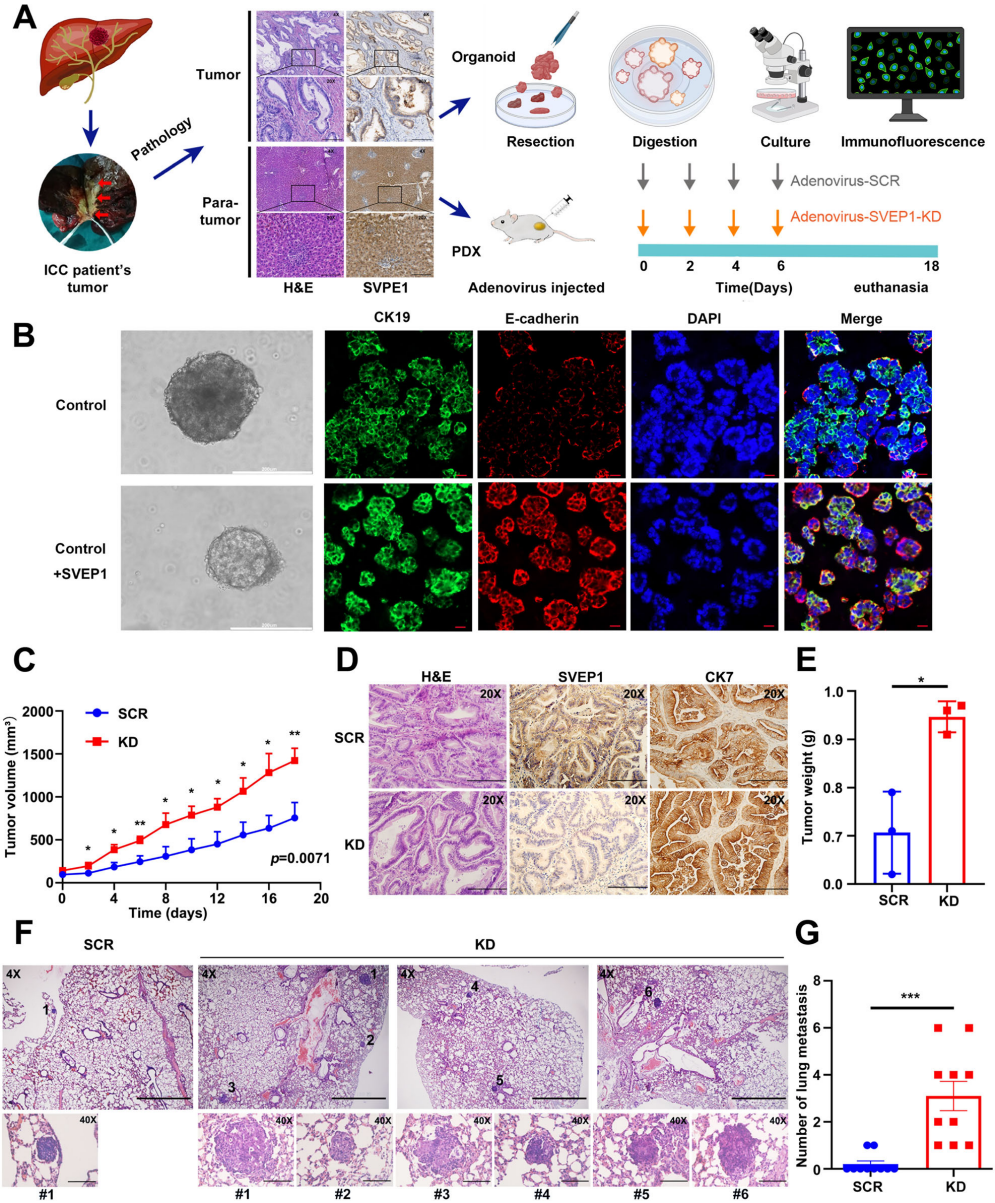
Suppressed expression of SVEP1 in ICC promotes tumor progression and metastasis in vivo. **A** Experimental design for in vivo studies using PDOs and PDX models. ICC tissues obtained from clinical patients undergoing surgical resection were used to establish ICC -PDOs and six ICC -PDX models. PDX models were divided into two groups (*n* = 3 per group). Adenovirus carrying SVEP1-KD or SCR was intratumorally injected, and tumor growth and weight were monitored. **B** Immunofluorescence staining of biliary marker (CK19, green) and epithelial marker (E-cadherin, red) with DAPI (blue) in patient-derived organoids treated with or without SVEP1-recombinant protein, shown with merged overlays (Scale bars, 200 μm and 20 μm). **C** Tumor growth curves and volume over time in SCR and KD groups of mice. **D** IHC staining validating SVEP1 knockdown in ICC -PDX models following adenovirus-SVEP1-KD injection in vivo (Scale bars, 100 μm). **E** Comparative analysis of tumor weights between two groups of mice. Representative images (**F**) and statistical analysis (**G**) of pulmonary metastatic nodules in the RBE group transfected with SVEP1 or control shRNAs using a lung metastasis model (Scale bars, 400 μm or 50 μm). *p < 0.05, **p < 0.01, ***p < 0.001.

Next, we further investigated the role of SVEP1 in regulating ICC cell differentiation and phenotype transition, as well as mediating tumor progression and metastasis using ICC PDOs and PDX models (Fig. 5A). A significant increase was observed in E-cadherin expression in ICC patient-derived organoids (PDOs) treated with the SVEP1-recombinant protein compared to the control group. This finding indicates that restoring SVEP1 is critical for maintaining the cellular epithelial phenotype in ICC (Fig. 5B).

Based on these findings, we hypothesized that SVEP1 depletion in ICC contributes to the loss of the cellular epithelial phenotype and promotes tumor progression and metastasis in vivo. To test this hypothesis, a mouse PDX ICC model was established. Figure 5A depicts that six ICC-PDX mice were divided into two groups and intra-tumorally injected every alternate day with Adenovirus-SVEP1-KD or its control counterpart when the tumors reached a certain volume (100 mm^3^). Tumor growth was monitored at specific time points, revealing marked differences between the two groups (Fig. 5C). By the 18th day, one ICC-PDX mouse succumbed to an excessive tumor burden, prompting the simultaneous euthanasia of the remaining five mice to assess tumor weight between the two groups. Consistent with previous study findings, SVEP1 inhibition resulted in significantly heavier and larger tumors in the Adenovirus-SVEP1-KD group than in the Adenovirus-SVEP1-SCR group (Fig. 5D, E).

To explore the effect of SVEP1 depletion on ICC metastasis in vivo, we established a pulmonary metastasis model, using SVEP1/KD and SVEP1/SCR RBE cells via tail vein injection (*n* = 10 mice per group). Metastatic nodules in the lungs were identified and quantified via H&E staining. Subsequently, two pathologists evaluated the nodules independently. Then, we selected a representative mouse per group (SCR and KD) exhibiting the highest metastatic burden under microscopic examination as representative cases, and comprehensively exhibited all microscopically detectable metastatic lesions of this mouse (Fig. 5F). Consistent with clinical analyses and in vitro experimental results, SVEP1 downregulation in RBE cells enhanced their metastatic ability in vivo. Figure 5G shows that the average number of lung metastatic nodules per mouse in the SVEP1/KD RBE group was 3.1, which was significantly higher than that in the control group (0.2, *p* < 0.001). These findings collectively suggest that SVEP1 expression has beneficial effects on maintaining the cellular epithelial phenotype and preventing tumor progression and metastasis in vivo.

### SVEP1 depletion in ICC mediated Jag2/Notch1/Hes5 signaling axis activation

**Fig. 6:**
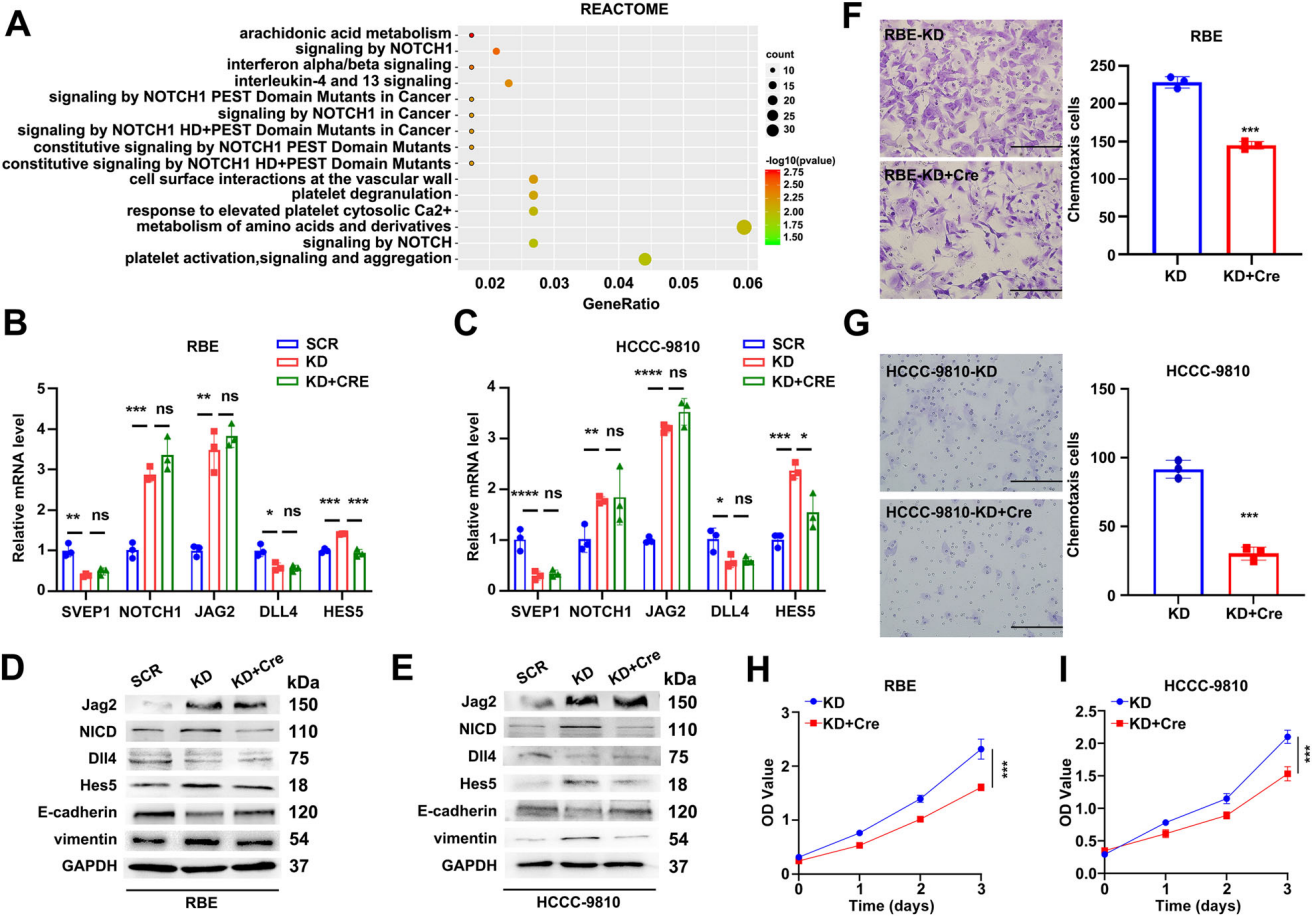
SVEP1 depletion in ICC mediates Notch1 activation. **A** Reactome signal pathway enrichment analysis was performed on significant DEGs obtained from RNA-seq profiling of SVEP1 KD vs. SVEP1 SCR. Bubble colors represent the adjusted *p*-value (red = most significant). The rich factor represents the proportion of enriched genes for each term. Quantitative RT-PCR analysis validates the effect of SVEP1 depletion and crenigacestat addition on the mRNA level of Notch1 pathway-related genes (NOTCH1, JAG2, DLL4, HES5) in RBE (**B**) and HCCC-9810 (**C**) cells. WB analysis of the inhibition of SVEP1 expression deficiency in RBE (**D**) and HCCC-9810 (**E**) cells on Notch1 pathway activation and EMT-phenotype switching, following the addition of crenigacestat. Representative images show the chemotactic migrating of RBE (**F**) and HCCC-9810 (**G**) cells transfected with SVEP1 shRNA and inhibited Notch1 following the addition of crenigacestat. Analysis of cell proliferation using CCK-8 assay in RBE (**H**) and HCCC-9810 (**I**) cells transfected with SVEP1 shRNA treated with Cre (−) or Cre (+). Cre is short for crenigacestat (Notch1 inhibitor). ns non-significant, *p < 0.05, **p < 0.01, ***p < 0.001, ****p < 0.0001.

To explore the potential molecular mechanisms underlying SVEP1 depletion in EMT phenotype switching in ICC, we conducted functional enrichment analysis using the Reactome database based on DEGs between RBE/KD and RBE/SCR cells. Figure 6A shows the top 15 significantly different biological processes between these two groups, emphasizing enrichment in Notch1 and its associated pathways (Fig. 6A and Supplementary Table 6). Previous studies have highlighted dysregulation involving Notch1 and its ligands during ICC progression [31]. Additionally, abnormal *JAG2* expression in myoepithelial cells has been implicated in supporting oncogenic Notch signaling [32]. Building on these insights, several selected DEGs involved in the Notch1 signaling pathway between RBE/KD and RBE/SCR cells were validated through RT-PCR and WB analyses. SVEP1 depletion in RBE and HCCC-9810 cell lines increased the mRNA levels of *NOTCH1*, *JAG2*, and *HES5* while reducing *DLL4* mRNA levels compared to those of their respective control groups (Fig. 6B, C). Furthermore, *HES5* downregulation following crenigacestat (Notch1 inhibition) in RBE/KD and HCCC-9810/KD cells was observed. However, no significant changes in *JAG2* upregulation and *DLL4* downregulation in SVEP1-depleted cells were observed (Fig. 6B, C).

Notch1 inhibition through Hes5 has been reported to significantly suppress cell migration and EMT in anaplastic thyroid carcinoma [33]. Building on this knowledge, we investigated whether Notch1/Hes5 activation drives EMT phenotype switching in SVEP1-depleted ICC cells. We observed restored E-cadherin expression in SVEP1-depleted cells upon crenigacestat treatment. This treatment also led to reduced vimentin expression and lower NICD and Hes5 levels in the Notch1-inhibited group compared with those of the control group (Fig. 6D, E). Correspondingly, inhibiting the Notch1/Hes5 signaling pathway markedly reduced the enhanced chemotactic motility induced by SVEP1 downregulation in ICC cells (Fig. 6F, G). Additionally, the proliferation-promoting effect of SVEP1 depletion in RBE and HCCC-9810 cells was effectively counteracted by crenigacestat application (Fig. 6H, I).

These findings revealed that SVEP1 depletion disrupts Notch1 ligand binding by specifically upregulating Jag2 and downregulating Dll4. This leads to the activation of the Notch1 signaling pathway through Hes5, which is crucial for driving EMT phenotype switching of ICC, thereby promoting malignant cellular behaviors.

### Reduced binding of SVEP1/integrin α9β1 promoted the malignant transformation of ICC through Notch1 signaling pathway activation

**Fig. 7:**
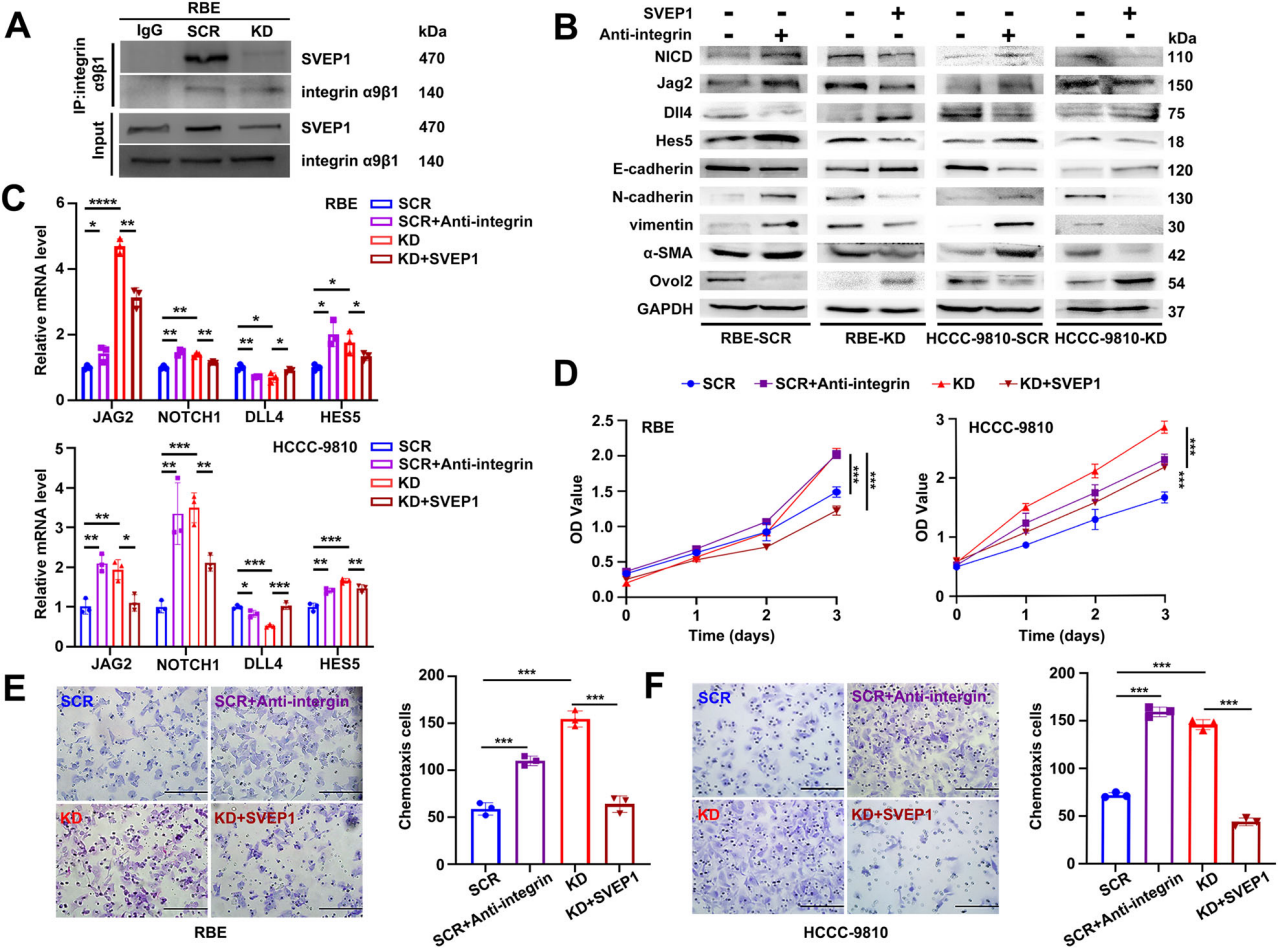
Notch1 activation depends on reduced binding of SVEP1/integrin α9β1. **A** WB analysis shows endogenous SVEP1 co-immunoprecipitating with endogenous integrin α9β1 in RBE cells. **B** WB analysis examines the effects of exogenously added integrin α9β1 antibody (Anti-integrin) in SCR cells and exogenous addition of SVEP1-recombinant protein (SVEP1) in SVEP1 KD cells on Notch1 pathway activation and EMT-phenotype switching, respectively. **C** RT-PCR analysis validates the influence of interfering with the binding of SVEP1/integrin α9β1 on Notch1 pathway activation by adding integrin α9β1 antibody to the SCR group or SVEP1-recombinant protein to the SVEP1 KD group. Analysis of CCK-8 proliferation assay (**D**) and representative images of chemotactic migrating assay (**E**, **F**) showed the influence of disrupting SVEP1/integrin α9β1 binding with integrin α9β1 antibody or SVEP1-recombinant protein on ICC cell malignant biological characteristics (Scale bars, 100 μm). *p < 0.05, **p < 0.01, ***p < 0.001, ****p < 0.0001.

These observations suggest that decreased SVEP1 expression could lead to the constitutive activation of Notch1 in ICC. To investigate the molecular mechanism underlying Notch1 signaling pathway activation owing to SVEP1 depletion, we conducted co-immunoprecipitation (Co-IP) experiments, consistent with previous studies [22], which indicated direct binding between SVEP1 and integrin α9β1 in RBE cells (Fig. 7A). Increased integrin α9β1 expression has been suggested as a novel marker linked to reduced survival in breast and lung cancer [34, 35]. However, its expression in CCA and its prognosis correlation remain unclear, as analysis of TCGA database indicated no abnormal increase or significant correlation with CCA prognosis (*p* = 0.87, Supplementary Fig. 3E, F).

Given that decreased SVEP1 expression promotes EMT phenotype switching of ICC, cell migration, and invasion in vitro and in vivo, we speculate that these effects stem from reduced SVEP1 binding to integrin α9β1. Treatment of RBE/SCR and HCCC-9810/SCR cells with an anti-integrin antibody, which competitively inhibits SVEP1/integrin α9β1 binding, significantly activated the Notch1 pathway (NICD, Jag2, and Hes5), and EMT-phenotype switching was subsequently observed (Fig. 7B). Conversely, the exogenous SVEP1-recombinant protein markedly inhibited the mesenchymal phenotype and Notch1 pathway activation in RBE/KD and HCCC-9810/KD cells, restoring the epithelial phenotype, consistent with previous study findings (Fig. 7B). RT-PCR analysis further confirmed that abnormal reduction of SVEP1 and integrin α9β1 binding activated the Jag2/Notch1/Hes5 signaling axis, which was attenuated by competitive binding with anti-integrin in the control group cells or rescued by treatment with SVEP1-recombinant protein in SVEP1-depleted cells (Fig. 7C). Furthermore, we observed that the SVEP1-recombinant protein attenuated the enhanced effects of SVEP1 depletion on RBE and HCCC-9810 cell proliferation and chemotactic migration (Fig. 7D–F). Conversely, treatment with anti-integrin in the control group cells significantly increased malignant biological behaviors, such as cellular proliferation and chemotactic migration (Fig. 7D–F).

**Fig. 8:**
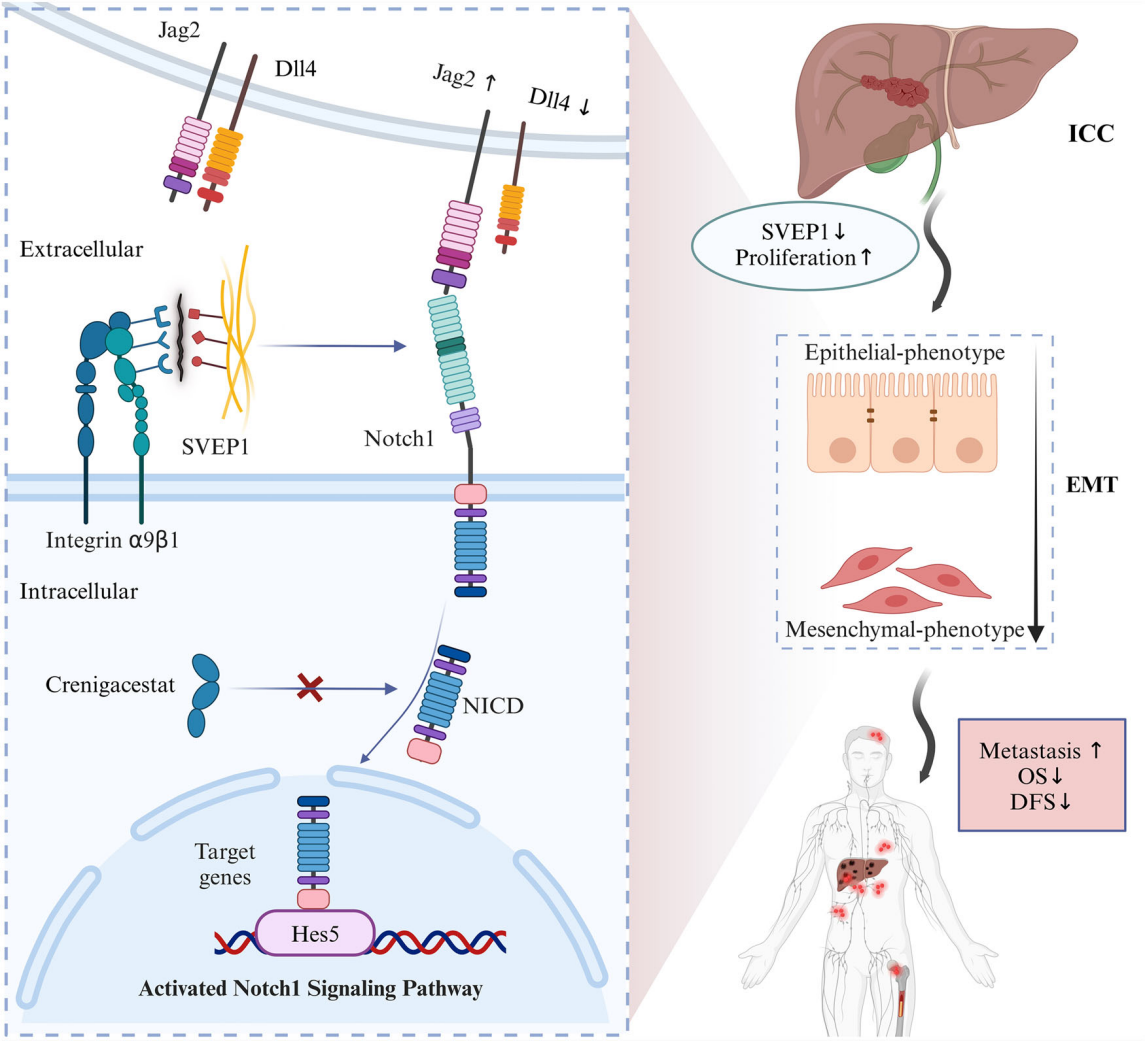
Schematic representation of the speculative molecular mechanism model. SVEP1 depletion in ICC tumor tissues decreases its binding with integrin α9β1, activating the Jag2/Notch1/Hes5 signaling pathway. This activation promotes cellular EMT-phenotype switching, leading to the promotion of malignant progression, distant metastasis, and recurrence of ICC. The model was created using BioRender (https://biorender.com).

These findings support the conclusion that reduced SVEP1/integrin α9β1 binding, resulting from SVEP1 depletion, activates the Jag2/Notch1/Hes5 pathway, thereby promoting cellular EMT-phenotype switching and malignant transformation in ICC (Fig. 8).

## Discussion

The prognosis of ICC remains extremely poor owing to limited biomarkers and available treatment options [36, 37]. Our study revealed that decreased SVEP1 expression in ICC correlates with SN and LN metastasis, as well as Ki-67 expression. Patients with ICC with low-SVEP1 expression also exhibited poorer prognosis, as evidenced by a shorter survival rate than those with high-SVEP1 expression. Moreover, downregulation of SVEP1 expression enhanced cellular proliferation, chemotactic migration, and invasion abilities in vitro and in vivo, which is consistent with our previous findings in HCC, where SVEP1 inhibition promoted multiple lung metastases and osteophagic invasion in mice [18].

A recent study reported that silencing SVEP1 activates the PI3K/AKT pathway, promoting colorectal cancer progression [38]. However, the molecular mechanisms underlying this regulation remain poorly understood. Our study revealed that depletion of SVEP1 in ICC cells increased malignant biological behaviors through the reduction of the SVEP1/integrin α9β1 complex, inducing cellular EMT phenotype switching via the activation of the Jag2/Notch1/Hes5 signaling axis.

Treatment decisions of ICC are ideally made by an expert multidisciplinary team, which selects patients for therapies like surgical resection, perioperative chemotherapy, liver-directed therapies, transplantation, and systemic therapy, including cytotoxic therapy, targeted therapy, and/or immunotherapy [4]. The high heterogeneity across omics and histopathological levels significantly limits the effectiveness of ICC treatments [39]. Our group first identified abnormal SVEP1 expression alongside four other genes as high-heterogeneity prognostic biomarkers for HCC in 2022, based on large-scale single-cell sequencing of samples from patients with varied recurrence characteristics [19]. However, the potential regulatory mechanisms underlying the abnormal expression of these genes and tumor heterogeneity warrant further exploration.

Numerous studies suggest that tumor cell phenotype switching may be a key factor in tumor heterogeneity and poor prognosis [40]. This switch enables tumor cells to temporarily change their morphological and functional characteristics during cancer development, allowing them to proliferate under harsh conditions and acquire invasive, metastatic, and drug-resistant phenotypes [41, 42]. For instance, Sacchetti et al. identified a subpopulation of phenotypically plastic colon cancer cells, EpCAM^lo^ cells, exhibiting hybrid E/M states (partial EMT), making them motile, invasive, chemo-resistant, and highly metastatic [43]. SVEP1 is thought to be involved in cell adhesion, migration, and motility [44]. Specifically, SVEP1 has been reported to regulate monocyte/macrophage differentiation phenotypes through an integrin α4β1/α9β1-dependent mechanism [45]. However, whether SVEP1 participates in the process of tumor cell phenotype switching remains unknown.

Integrins, a ubiquitous family of cell membrane adhesion receptors, contribute to the ECM with two main functions: providing mechanical connections to the ECM and activating signal transduction pathways, which regulate various cellular functions necessary for the initiation, progression, and metastasis of solid tumors [46, 47]. Epithelial-mesenchymal transition (EMT) is a key regulator of metastasis, conferring an invasive phenotype [48]. During tumorigenesis, E-cadherin loses its function, transitioning to a motile and invasive phenotype in concert with integrin-mediated adhesion to the surrounding ECM [49]. Previous studies indicate that targeting integrin signaling reverses EMT in breast and oral cancer, among others [50, 51]. In the current study, we observed that the downregulation of SVEP1 in ICC led to the loss of the epithelial phenotype, as evidenced by decreased E-cadherin expression. Conversely, mesenchymal biomarkers such as vimentin, N-cadherin, and α-SMA increased, indicating that SVEP1-depleted cells acquired a mesenchymal phenotype and were transitioning from an epithelial to mesenchymal state. Furthermore, our findings revealed that the exogenous addition of SVEP1-recombinant protein might reverse EMT phenotype switching and weaken the malignant biological characteristics of SVEP1-depleted cells.

Recent studies have primarily focused on investigating the effects of Notch1 and its ligands on cancer metastasis and drug resistance. For example, high Jag1 expression plays an important role in mediating plasticity and tumor heterogeneity in pancreatic ductal adenocarcinoma cells [52]. Similarly, the activation of the Jag1/Notch1 pathway has been implicated as a key factor in the drug resistance of trastuzumab in breast cancer [53]. Notch1 activation also promotes ICC progression and correlates with chemoresistance [54]. However, the role of Notch1 and its binding ligands in regulating EMT phenotype switching has been rarely reported. Our study revealed the upregulation of Jag2, Notch1, and Hes5 in SVEP1-depleted ICC cells, along with the downregulation of Dll4. Jag2 promotes colon cancer cell migration and invasion through a non-EMT pathway independent of the canonical Notch signaling pathway [44]. However, our findings provide compelling evidence that Jag2 facilitates malignant cellular behavior by promoting EMT phenotype switching in a Notch1-dependent manner in ICC. This was shown in SVEP1-depleted cells treated with a Notch1 inhibitor, where, despite high Jag2 expression, the EMT process was reversibly halted. Consistent with these findings, activating the Jag2/Notch1/Hes5 signaling axis by interfering with SVEP1/integrin α9β1 binding significantly increased the proliferation, migration, and invasion of ICC cells.

Our study revealed SVEP1 as an independent prognostic factor for ICC, elucidating its role in regulating the malignant progression of this cancer. These findings reveal a novel molecular mechanism by which SVEP1 depletion induces tumor cell EMT phenotype switching, thereby enhancing our understanding of ICC progression, recurrence, and metastasis.

## Methods

### Patients and specimens

This study was approved by the Ethics Committee of Tianjin Medical University Cancer Institute and Hospital (No. Ek2023045), and informed consent was obtained from all participating patients. All samples were sourced from the Tianjin Medical University Cancer Institute and Hospital (Tianjin, China).

Overall, 113 paired samples of ICC and their corresponding para-tumor tissues were analyzed using immunohistochemistry (IHC). Additionally, 5-paired tissues were used for western blotting, and 10-paired tissues were used for quantitative real-time PCR.

Furthermore, 1 sample was used to construct patient-derived organoids (PDOs) and patient-derived xenograft (PDX) model. The clinical details of this ICC sample are as follows: the patient is a 60-year-old female, preoperative laboratory test: AFP level was 2.33 ng/ml, CA19-9 level was 76,745 U/ml. Histopathological examination of specimen revealed a moderately to poorly differentiated cholangiocarcinoma. The tumor was solitary, measuring 6 cm in maximum diameter, and was associated with vascular invasion. There was no evidence of lymph node metastasis. Immunohistochemical staining demonstrated positive expression for CK7 and CK19.

### Immunohistochemistry

IHC staining was utilized to assess SVEP1, E-cadherin, vimentin, and Ki-67 expression levels in paraffin-embedded samples from ICC and normal tissues based on previously described methods [9]. IHC was also utilized to assess the expression of SVEP1 and Cytokeratin (CK7) in tumor tissues of mouse PDX models. The following antibodies were used: anti-SVEP1 (1:200), anti-vimentin (1:500) (Abcam, Cambridge, England), anti-E-cadherin (1:100) (Sino Biological, China), anti-Ki-67 (1:100) (Roche, Switzerland), and anti-CK7 (1:100) (Abclonal Technology, China). IHC scoring criteria for SVEP1, E-cadherin, vimentin, and CK7 were as follows: staining intensity graded into four categories (0 = no staining, 1 = weak staining, 2 = moderate to strong staining, and 3 = strong staining). The percentage of positively stained cells was categorized into four levels (0 = 0% positive cells, 1 = <30% positive cells, 2 = 30–60% positive cells, and 3 = 60–100% positive cells). The final IHC score was calculated by multiplying the staining intensity score with the percentage score. The cutoff value for final IHC scores of SVEP1, E-cadherin, and vimentin was set at 6, with scores of ≥6 and <6 indicating high and low expressions, respectively. For Ki-67 intensity analysis, a percentage of positive tumor cells >20% was considered high, while that <15% was deemed low. This approach enabled a comprehensive assessment of protein expression levels and their correlation with clinical outcomes in patients with ICC.

### Cell culture

Human ICC cell lines RBE and HCCC-9810 were purchased from Feiouer Biotechnology Co., Ltd. The cells were cultured in RPMI 1640 medium (Corning, NY, USA) supplemented with 10% fetal bovine serum (FBS, PAN–Seratech) and 1% penicillin/streptomycin (Hyclone). For cell transfection, lentiviral particles were generated by transfecting packaging plasmids (VSVG and ΔR) and expression plasmids (SVEP1-knockdown (KD) and matched scramble (SCR)) into HEK293T cells using Lipofectamine 3000 (Invitrogen). Subsequently, the cells were infected with the lentivirus to establish stable SVEP1-KD or SCR cell lines. Stably transfected cell lines were selected using puromycin (Gibco) at a concentration of 0.4 µL per 1 mL of culture medium.

**Table:** 

shRNA used to generate SVEP1 KD clones
**Clone1-KD**5′-CCGGATCGATTCTAAGAGCATATTTCTCGAGAAATATGCTCTTAGAATCGATTTTTT-3′
**Clone2-KD2**5′-CCGGTTAGGGTAGCCTGGCTAATTCCTCGAGGAATTAGCCAGGCTACCCTAATTTTT-3′

For the crenigacestat (Selleck Chemicals LLC, Houston, USA) treatment, cells are inoculated at a concentration of 5 × 10^5^ cells per well in a six-well plate. The SVEP1 knockdown (KD) group is subjected to treatment with or without 10 µM crenigacestat for a duration of 24 h. Subsequent to the treatment period, cells are collected for the extraction of proteins or RNA, which will be utilized for subsequent experiments.

For treatments involving SVEP1 recombinant protein (Bio-techne China Co., Ltd., Shanghai, China) and integrin α9β1 blockade (Abcam, Cambridge, England), cells were similarly seeded at a density of 5 × 10^5^ cells per well in a six-well plate. After allowing the cells to adhere for 12 h, the scramble (SCR) group was treated with or without 10 µM anti-integrin α9β1 antibody, while the KD group was treated with or without 2 µg of SVEP1 recombinant protein for 24 h, respectively. And the cell pellets were collected for use in subsequent experimental analyses.

### Western blotting

Freshly frozen ICC tissues were extracted using a tissue protein extractor (Service Bio, Wuhan, China). Western blotting (WB) was employed to compare SVEP1 expression levels in tumor and para-tumor tissues. Cells were washed thrice with cold phosphate-buffered saline (PBS) and lysed on ice for 30 min using SDS lysis buffer supplemented with 1 mM NaF, 1 mM Na_3_VO_4_, and a 1× protease/phosphatase inhibitor cocktail (Roche, Switzerland). The collected protein was denatured in a 95 °C-metal bath for 10 min and centrifuged at 12,000 rpm at 4 °C for approximately 10 min. Equal amounts of protein were loaded onto the gel and separated by sodium dodecyl sulfate-polyacrylamide gel electrophoresis (SDS-PAGE) using an 8% or 10% gel. Subsequently, the proteins were loaded onto a polyvinylidene fluoride (PVDF) membrane (Immobilon-P; Millipore, MA, USA) and blocked with 5% skim milk or 3% bovine serum albumin (BSA). The membrane was then incubated with primary antibodies against the following targets: SVEP1 (1:250, R&D Systems), Notch1 (1:1000), Jag2 (1:1000), and Dll4 (1:500) from Cell Signaling Technology (Beverly, USA); Hes5 (1:800) from Bioss; E-cadherin (1:1000) from BD Biosciences (San Jose, USA); GAPDH (1:1000), ZEB1 (1:100), Ovol2 (1:200), and N-cadherin (1:400) from Santa Cruz Biotechnology (Santa Cruz, USA); vimentin (1:8000) from Epitomics (Burlingame, USA); α-SMA (0.2 mg/mL), NICD (1:500), integrin α9β1 (1:1000), and Twist (1:1000) from Abcam (Cambridge, England). Finally, the membrane is incubated with secondary antibodies against the following targets: m-IgGκBP-HRP (1:4000) and mouse anti-rabbit IgG-HRP (1:4000) from Santa Cruz Biotechnology (Santa Cruz, USA).

### Co-Immunoprecipitation

The cell lysates were incubated overnight at 4 °C with the antibodies on a rotator. Protein A/G Dynabead complexes (30 µL, Life Technologies, USA) were prepared. Subsequently, a normal IgG control was included in the assay. After centrifugation to pellet the Dynabeads, the resulting supernatants underwent SDS-PAGE and immunoblotting. The immunocomplexes were then analyzed using WB analysis.

### Chemotaxis assay

For the chemotaxis assay, 24-well plates and 8-μm-pore chemotaxis chambers (Falcon Cell Culture Inserts, Corning, USA) were utilized. Overall, 1 × 10^5^ cells in 200 μL of 1640 medium with 10% FBS were seeded into the upper chamber, while 600 μL of culture medium containing 20% FBS was added to the lower chamber. After 6 h of incubation, the Transwell chambers were fixed with 4% paraformaldehyde for 10 min, followed by activation in 100% methanol for an additional 10 min. The cells were then washed, fixed, and stained. Cells that migrated through the membrane were counted under a microscope for comparison.

### Invasion assay

Cell invasion ability was assessed using a transwell assay. Matrigel (1 mg/mL) (BD Biocoat, Corning, NY, USA) was diluted according to the instructions of the manufacturer and added to the upper chamber of the Transwell plates. In total, 1 × 10^5^ cells were suspended in 1640 medium with 2% FBS and seeded into the upper chamber, while the lower chamber received 600 μL of 1640 medium supplemented with 20% FBS. After 9 h of incubation, the Transwell chambers were fixed with 4% paraformaldehyde for 10 min, followed by activation with 100% methanol for another 10 min. The cells were then stained, washed thrice with PBS, and allowed to dry. Cells that invaded through the Matrigel and membrane were counted under a microscope for comparison.

### Wound-healing assay

For the wound-healing assay, cells were digested, centrifuged, and resuspended to create a cell suspension. Subsequently, (1.5–2) × 10^6^ cells were seeded in a 6-well plate. The next day, a 10 μL pipette tip was used to create a uniform wound in each well after the cells had adhered. The wells were then washed twice with PBS, and Dulbecco’s Modified Eagle medium (DMEM) supplemented with 2% FBS was added. Cells were then photographed under an inverted microscope (100 × magnification) at 0, 12, and 24 h to document their movements. Wound closure was quantified as follows: Closure/Start_wound% = ((0 h wound area – 12 h (or 24 h) wound area) × 100)/0 h wound area.

### Plate colony formation assay

Cells with the specified genetic manipulations were seeded into 6-well tissue culture plates at a density of 1000 cells per well. After 10–14 days, the colonies were fixed with 100% methanol, stained with 0.1% crystal violet, imaged, and counted, and the data are presented as the means ± SDs of triplicate dishes in the same experiment.

### Cell counting kit-8 (CCK-8) assay

Cell viability was assessed using the CCK-8 assay. Briefly, 2 × 10^3^ cells in 100 μL of 1640 medium were seeded per well in a 96-well plate. Following treatment with appropriate reagents, 10 μL of CCK-8 solution (Biosharp Life Sciences, Beijing, China) was added to each well and incubated for 2 h at 37 °C. The optical density (OD) was then measured at 450 nm using a microplate reader, and cell proliferation curves were generated by continuously monitoring for 3–4 days.

### Immunofluorescence

Sterile coverslips were placed in 12-well plates, with 4 × 10^4^ cells seeded into each well. After incubating at 37 °C in 5% CO_2_ for 12 h, the cells were fixed in 4% paraformaldehyde, permeabilized using 2% Triton X-100, and blocked with 3% BSA at 25 °C for 1 h. Subsequently, cells were incubated overnight at 4 °C with primary antibodies against SVEP1 (1:200, Abcam, Cambridge, England), E-cadherin (1:20, BD Biosciences, San Jose, USA), and vimentin (1:5000, Epitomics, Burlingame, USA). After washing twice with PBS, the cells were stained with an Alexa Fluor 488-conjugated secondary antibody (Invitrogen, Carlsbad, USA) at 25 °C for 1 h in the dark. Cell nuclei were counterstained using ProLong Gold Antifade Reagent (Invitrogen, Carlsbad, USA). Imaging was captured using a confocal laser-scanning microscope (Olympus FV1000).

### Quantitative real-time PCR (RT-PCR)

Total RNA was isolated from cells and mouse livers using TRIzol reagent (Ambion, USA) and evaluated using a NanoDrop 2000 spectrophotometer (Thermo Scientific, Pittsburgh, USA). Then, the One-Step RNA PCR Kit (AMV) from TaKaRa Biotechnology Co., Ltd (Dalian, China) was used to transcribe RNA. The amplification reaction needed 35 cycles. Each cycle contained denaturation at 95 °C for 1 min, annealing for 45 s, and an extension at 72 °C for 1 min. A final extension step at 72 °C for 5 min terminated the amplification. Supplementary Table 1 depicts the sequences of all primers.

### Organoid culture and passage

The ICC tissue was carefully cut into small fragments (approximately 0.5–1 mm in diameter) using precision dissection scissors and rinsed three times with cold phosphate-buffered saline (PBS) at 4 °C in 50 mL Falcon tubes. Dissociation of the tissue was performed using the Tumor Dissociation Kit (jiyanbio, Shanghai, China) as per the manufacturer’s instructions. Incubation at 37 °C was carried out for 30 to 90 min, depending on the tissue volume, until the majority of cells were released into suspension. To stop the digestion, cold DMEM containing 10% FBS was added. The resulting cell suspension was passed through a 70 μm nylon cell strainer and centrifuged at 500 × *g* for 5 min. Red blood cells were removed by incubating the cells with 1× RBC lysis buffer (Solarbio, Beijing, China) under gentle rotation at 4 °C for 10 min. Following removal of the lysis buffer, the cells were combined with 30% growth factor-reduced Matrigel (Corning, NY, USA) and plated in ultra-low-attachment 24- or 48-well plates, depending on the number of viable cells. After 30 min, once the Matrigel had solidified, warm ICC organoid isolation culture medium (jiyanbio, Shanghai, China) was added. Culture medium was replenished every 2 days.

For passaging, ICC organoids were broken into smaller fragments using a P1000 pipette. The fragments were washed with 10 ml of culture medium, centrifuged at 200 × *g* for 2 min, and resuspended in Matrigel at a 1:5 dilution ratio. For long-term storage, organoid cultures were dissociated into single cells or small pieces and frozen in Recovery Cell Culture Freezing Medium (Invitrogen, Carlsbad, USA) at -80°C or in liquid nitrogen. Cryopreserved stocks remained viable and could be successfully recovered for up to 2 years. After confirming the successful construction of the organoids, 2 µg of SVEP1 recombinant protein addition and histological analyses were conducted. Briefly, sections were dewaxed in xylene, rehydrated using an alcohol gradient, and incubated with serum. The sections were then incubated with primary antibodies (E-ca: 1:20, BD Biosciences, San Jose, USA; CK19:1:100, Zhongshan Chemical Co, Zhongshan, China), followed by secondary antibodies. Finally, DAPI was used to stain the nuclei.

### PDX model

Animal experiments were approved by the Ethics Committee of Tianjin Medical University Cancer Institute and Hospital (No.NSFC-AE-2023037). Five-week-old male NSG (NOD.Cg-Prkdc^scid^ IL2rg^em1Smoc^) mice (Shanghai Model Organisms Center, Inc., Shanghai, China) were used for the xenograft animal experiments. Fresh patient ICC specimens were minced into 2 × 2 mm fragments. P1 tumors were established by subcutaneously implanting these fragments into the flanks of nude mice. Once P1 tumors developed, they were harvested, re-sectioned into 2 × 2 mm pieces, and passaged subcutaneously to generate P2 tumors. After P2 tumors stabilized, the fragments were again randomized and implanted into the flanks of experimental mice. When the tumors reached a volume of 100 mm³, the mice were administered intra-tumoral injections of Adenovirus-SVEP1-KD or Adenovirus-SCR (1 × 10^9^ Pfu in a 100 μL volume) every other day. Tumor growth was monitored at the specified time points. Informed consent was obtained from the patients or their relatives, and the study was approved by the Ethics Committee of Tianjin Medical University Cancer Institute and Hospital (No.Ek2023045).

### Metastasis assay in vivo

Animal experiments were approved by the Ethics Committee of Tianjin Medical University Cancer Institute and Hospital (No.NSFC-AE-2023037). Five-week-old nude mice (SPF Biotechnology Co., Ltd., Beijing, China) were used for the xenograft animal experiments. To generate lung metastasis models, RBE cells transfected with SVEP1 or control siRNAs (2 × 10^6^/mL; 100 μL/mice) were prepared and injected into the tail vein of each mouse (10 mice/group). After six weeks, the mice were euthanized, and the lungs were harvested. The harvested lung tissues were then formalin-fixed, paraffin-embedded, and stained with hematoxylin and eosin. Randomization was used to allocate the animals to the experimental groups and process them, with the investigator blinded to the group allocation during the experiment.

### mRNA sequencing analysis

Total mRNA was isolated from RBE/KD and RBE/SCR cells. Quality assessment of 150 bp paired-end reads was conducted using FastQC (v0.12.1), and quantification estimation was conducted using Salmon (v1.10.1) based on the gene annotation for human build hg38 obtained from GENCODE (release 45). Differential gene expression analysis was conducted using DESeq2 (v1.44.0) and salmon quantification results and gene annotations. Genes with |log2 (Fold Change)| ≥ 1 and adjusted *p* value ≤ 0.05 were filtered as differentially expressed genes (DEGs). To identify DEGs distinct to the high-recurrence group, a two-step approach was employed. Initially, DEGs were identified by comparing tumor tissues with the corresponding adjacent normal tissues in high- and low-recurrence groups. Subsequently, only the DEGs exclusive to the high-recurrence group were selected for further analysis. Gene ontology and Kyoto Encyclopedia of Genes and Genomes (KEGG) pathway enrichment analysis were conducted using ClusterProfiler (v4.10.0) with an enrichment cutoff of FDR ≤ 0.05. Protein-protein interaction data from the STRING database (v12.0) were utilized to construct the interaction network. The RNA-Seq data used in this study can be obtained from the corresponding author upon request.

### Gene set enrichment analysis

Gene set enrichment analysis (GSEA) is an analytical method for studying genome-wide expression profiles from microarray data. It identifies functional enrichment by comparing genes with predefined gene sets. GSEA was conducted to assess the relationship between SVEP1 mRNA levels and prognosis, as well as biological states such as proliferation, epithelial-mesenchymal transition, and high expression of some genes in tumors. This analysis was based on datasets GSE132305, GSE89749, and GSE45001 for CCA, employing GSEA 4.3.3 software developed by The Broad Institute of MIT and Harvard.

### Statistical analysis

All data are presented as means ± SDs. Statistical analyses were conducted using SPSS 26.0 (SPSS Inc., Chicago, IL, USA) and GraphPad Prism 9.5 (GraphPad Software, Inc.). Survival curves were plotted using the Kaplan–Meier method and analyzed using the log-rank test. Group differences were evaluated using a two-tailed Student’s *t*-test or analysis of variance (ANOVA), where relevant. Pearson’s correlation test was employed for correlation analysis. A *p*-value of <0.05 was considered statistically significant.

## Supplementary information

Supplementary tables

Supplementary figure legends

Supplementary figures

Figure S1

Figure S2

Figure S3

WB-RAWDATA

## Data Availability

All the original data presented in this study are included in the article and supplementary material. Any further requests can be directed to the corresponding authors.
