# Supplementary material for: Low SVEP1 in intrahepatic cholangiocarcinoma mediates phenotype switching-driven metastasis by Jag2/Notch1/Hes5
Source: Cell Death Dis. 2025 Nov 28;16(1):871. doi: 10.1038/s41419-025-08170-2 (PMC12663138; doi:10.1038/s41419-025-08170-2)
Supplement: Supplementary file 1 — Supplementary tables [file 41419_2025_8170_MOESM1_ESM.docx]

**Supplementary Table 1. qRT-PCR primers of mRNA mRNA primer sequences**

Human *SVEP1* Forward Primer GCAAACCACGGGTTCAAGTC Human *SVEP1* Reverse Primer CCAGGGTCGTCTCAAATGCT Human *GAPDH* Forward Primer TGGTATCGTGGAAGGACTCA Human *GAPDH* Reverse Primer CCAGTAGAGGCAGGGATGAT Human *CDH1* Forward Primer CACACGGGGCGAGTGCCAAC Human *CDH1* Reverse Primer GCGGCCCCTTCACAGTCACA Human *CDH2* Forward Primer AGCCAACCTTAACTGAGGAGT Human *CDH2* Reverse Primer GGCAAGTTGATTGGAGGGATG Human *OVOL1* Forward Primer CACGTGCAAGAGGAACTGGA Human *OVOL1* Reverse Primer GTTCGGCCACAGAGGGTT Human *OVOL2* Forward Primer TCACCTCAAGTGCCACAACCA Human *OVOL2* Reverse Primer TGTAGCCGCAATCCTCGCA Human *OCLN* Forward Primer CACACTTGCTTGGGACAGAGG

Human *OCLN* Reverse Primer TGAGCCGTACATAGATCCAGGAGC Human *VIM* Forward Primer GATGCCCTTAAAGGAACCAATGAG Human *VIM* Reverse Primer GGCGGCCAATAGTGTCTTGGTAG Human *SNAIL* Forward Primer CTGGGTGCCCTCAAGATGCA Human *SNAIL* Reverse Primer CCGGACATGGCCTTGTAGCA Human *SLUG* Forward Primer TACCGCTGCTCCATTCCACG

Human *SLUG* Reverse Primer CATGGGGGTCTGAAAGCTTGG Human *TWIST* Forward Primer GGAGTCCGCAGTCTTACGAG Human *TWIST* Reverse Primer TCTGGAGGACCTGGTAGAGG Human *ZEB1* Forward Primer TTCAAACCCATAGTGGTTGCT Human *ZEB1* Reverse Primer TGGGAGACACCAAACCAACTG Human *ZEB2* Forward Primer CGGTATTGCCAACCCTCTGGA Human *ZEB2* Reverse Primer TTGTTGTGCCAGGGGTGTTCC Human *NOTCH1* Forward Primer GCTGCCTCTTTGATGGCTTCGA Human *NOTCH1* Reverse Primer CACATTCGGCACTGTTACAGCC Human *JAG2* Forward Primer AACGATACCACCCCGAATGAGG Human *JAG2* Reverse Primer GCTGCCACAGTAGTTCAGGTCTTTG Human *DLL4* Forward Primer TTGGATGAGCAAACCAGCACCC Human *DLL4* Reverse Primer TGACAGCCCGAAAGACAGATAGG Human *HES5* Forward Primer AAGCACAGCAAAGCCTTCGT Human *HES5* Reverse Primer TGTCCTAAAACGGCAGGGAC

**Supplementary Table 2. Clinicopathological charactoristics of ICC patients for high-throughput RNA sequencing**

| **Number** | **gender** | **Age** | **Tumor Size** | **Lymph nodes** | **Distant Metastasis** | **TNM stage** | **Group** |
| --- | --- | --- | --- | --- | --- | --- | --- |
| 1 | male | 58 | 4.5 | 0 | 0 | T1aN0Mx | High-recurrence |
| 2 | male | 64 | 6 | 0 | 0 | T1bN0Mx | High-recurrence |
| 3 | male | 59 | 4 | 0 | 0 | T1aN0Mx | High-recurrence |
| 4 | male | 59 | 6.5 | 0 | 0 | T1bN0Mx | High-recurrence |
| 5 | male | 61 | 4 | 0 | 0 | T1aN0Mx | High-recurrence |
| 6 | male | 58 | 3.5 | 0 | 0 | T1aN0Mx | High-recurrence |
| 7 | male | 45 | 4 | 0 | 0 | T1aN0Mx | High-recurrence |
| 8 | male | 49 | 3 | 0 | 0 | T1aN0Mx | Low-recurrence |
| 9 | male | 55 | 3.5 | 0 | 0 | T1aN0Mx | Low-recurrence |
| 10 | male | 52 | 2.5 | 0 | 0 | T1aN0Mx | Low-recurrence |
| 11 | male | 57 | 6 | 0 | 0 | T1bN0Mx | Low-recurrence |
| 12 | male | 49 | 7 | 0 | 0 | T1bN0Mx | Low-recurrence |
| 13 | male | 48 | 4.5 | 0 | 0 | T1aN0Mx | Low-recurrence |
| 14 | male | 53 | 4 | 0 | 0 | T1aN0Mx | Low-recurrence |
| 15 | male | 70 | 4 | 0 | 0 | T1aN0Mx | Low-recurrence |
| 16 | male | 57 | 3.5 | 0 | 0 | T1aN0Mx | Low-recurrence |
| 17 | male | 55 | 6 | 0 | 0 | T1bN0Mx | Low-recurrence |

**Supplementary Table 3. Univariate and multivariate analysis of prognostic factors associated with DFS and OS in 113 ICC patients**

| **Univariate Analysis (DFS) Multivariate Analysis (DFS) Univariate Analysis (OS) Multivariate Analysis (OS)** | | | | | | | | | | | | | |
| --- | --- | --- | --- | --- | --- | --- | --- | --- | --- | --- | --- | --- | --- |
| **ICC patients** | **(n=113)** | **HR**  **(log rank)** | **95% CI** | ***p*-value** | **HR**  **(log rank)** | **95% CI** | ***p*-value** | **HR**  **(log rank)** | **95% CI** | ***p*-value** | **HR**  **(log rank)** | **95% CI** | ***p*-value** |
| **Age (years)** ≥55/<55 | | 1.268 | 0.791-2.034 | 0.323 |  |  |  | 1.179 | 0.665-2.091 | 0.573 |  |  |  |
| **Sex** male/female | | 0.795 | 0.514-1.229 | 0.300 |  |  |  | 1.044 | 0.608-1.793 | 0.876 |  |  |  |
| **CA19-9 (U/mL)** ≥27/<27 | | 1.991 | 1.242-3.192 | **0.004*** | 1.759 | 0.963-3.214 | 0.066 | 1.744 | 1.461-5.155 | **0.001*** | 2.131 | 0.949-4.786 | 0.067 |
| **CA242 (IU/mL)** ≥20/<20 | | 1.760 | 1.130-2.741 | **0.011*** | 1.065 | 0.603-1.881 | 0.828 | 2.116 | 1.230-3.641 | **0.006*** | 1.372 | 0.664-2.838 | 0.393 |
| **CEA (μg/mL)** ≥5/<5 | | 1.768 | 1.092-2.862 | **0.019*** | 1.160 | 0.674-1.997 | 0.592 | 1.627 | 0.903-2.931 | 0.102 |  |  |  |
| **TBIL (μmol/mL)** ≥21/<21 | | 2.489 | 1.528-4.056 | **<0.001*** | 3.664 | 1.977-6.788 | **<0.001*** | 2.024 | 1.133-3.613 | **0.015*** | 2.255 | 1.224-4.154 | **0.009*** |
| **DBIL (μmol/mL)** ≥8/<8 | | 1.519 | 0.780-2.956 | 0.215 |  |  |  | 1.171 | 0.516-2.657 | 0.706 |  |  |  |
| **IHC score of Ki-67** 0.940 0.606-1.459 0.784 0.694 0.397-1.212 0.196 | | | | | | | | | | | | | |
| ≥15%/<15% | |  |  |  |  |  |  |  |  |  |  |  |  |
| **Hepatitis** Y/N | | 0.999 | 0.584-1.70- | 0.998 |  |  |  | 1.123 | 0.600-2.102 | 0.717 |  |  |  |
| **Tumor size (cm)** >5/≤5 | | 0.984 | 0.576-1.680 | 0.952 |  |  |  | 0.655 | 0.326-1.316 | 0.231 |  |  |  |
| **Tumor number** >1/≤1 | | 1.734 | 1.000-3.010 | **0.047*** | 1.538 | 0.859-2.756 | 0.148 | 0.925 | 0.449-1.905 | 0.832 |  |  |  |
| **Microvascular invasion** Y/N | | 0.599 | 0.083-4.314 | 0.607 |  |  |  | 0.970 | 0.133-7.072 | 0.976 |  |  |  |
| **Tumor thrombus** Y/N | | 1.354 | 0.800-2.291 | 0.257 |  |  |  | 2.171 | 1.177-4.003 | **0.011*** | 2.691 | 1.358-5.222 | **0.005*** |
| **Lymph nodes metastasis** Y/N | | 1.738 | 1.037-2.911 | **0.033*** | 0.413 | 0.125-1.370 | 0.148 | 2.057 | 1.126-3.758 | **0.016*** | 1.529 | 0.485-4.822 | 0.469 |
| **Satellite nodule** Y/N | | 2.115 | 1.186-3.772 | **0.009*** | 4.277 | 1.442-12.686 | **0.009*** | 1.498 | 0.730-3.074 | 0.267 |  |  |  |
| **TNM stage** I&II/III&IV | | 1.664 | 1.047-2.645 | **0.029*** | 1.140 | 0.518-2.509 | 0.746 | 1.769 | 1.007-3.107 | **0.044*** | 1.240 | 0.418-3.683 | 0.698 |
| **IHC score of SVEP1** ≥6/<6 | | 0.535 | 0.316-0.906 | **0.018*** | 0.373 | 0.198-0.703 | **0.002*** | 0.263 | 0.112-0.617 | **0.001*** | 0.196 | 0.078-0.489 | **<0.001*** |

**Supplementary Table 4. Correlation between clinicopathological characteristics and SVEP1 expression in 113 ICC patients**

| **Characteristics Total SVEP1 *P*-value Characteristics Total SVEP1 *P*-value expression expression** | | | | | | | | | | | |
| --- | --- | --- | --- | --- | --- | --- | --- | --- | --- | --- | --- |
|  |  |  | **Low** | **High** |  |  |  |  | **Low** | **High** |  |
|  |  | **113** |  |  |  |  |  | **113** |  |  |  |
| **Age(years)** |  |  |  |  | 0.609 | **Tumor size (cm)** |  |  |  |  | 0.766 |
|  | ≥55 | 76 | 54 | 22 |  |  | >5 | 24 | 18 | 6 |  |
|  | <55 | 37 | 28 | 9 |  |  | ≤5 | 89 | 64 | 25 |  |
| **Sex** |  |  |  |  | 0.468 | **Tumor number** |  |  |  |  | 0.268 |
|  | Male | 61 | 46 | 15 |  |  | >1 | 18 | 15 | 3 |  |
|  | Female | 52 | 36 | 16 |  |  | ≤1 | 95 | 67 | 28 |  |
| **Hepatitis** |  |  |  |  | 0.943 | **Microvascular invasion** |  |  |  |  | 0.475 |
|  | Present | 25 | 18 | 7 |  |  | Present | 2 | 1 | 1 |  |
|  | Absent | 88 | 64 | 24 |  |  | Absent | 111 | 81 | 30 |  |
| **Tumor**  **thrombus** |  |  |  |  | 0.497 | **Satellite nodule** |  |  |  |  | **0.041*** |
|  | Present | 23 | 18 | 5 |  |  | Present | 16 | 15 | 1 |  |
|  | Absent | 90 | 64 | 26 |  |  | Absent | 97 | 67 | 30 |  |
| **CA19-9 (U/mL)** |  |  |  |  | 0.930 | **Lymph nodes metastasis** |  |  |  |  | **0.042*** |
|  | ≥27 | 70 | 51 | 19 |  |  | Present | 21 | 19 | 2 |  |
|  | <27 | 43 | 31 | 12 |  |  | Absent | 92 | 63 | 29 |  |
| **CA242 (IU/ml)** |  |  |  |  | 0.329 | **IHC score of Ki-67** |  |  |  |  | **0.003*** |
|  | ≥20 | 41 | 32 | 9 |  |  | ≥15% | 51 | 44 | 7 |  |
|  | <20 | 72 | 50 | 22 |  |  | <15% | 62 | 38 | 24 |  |
| **CEA (μg/mL)** |  |  |  |  | 0.057 | **IHC score of E-cadherin** |  |  |  |  | **0.011*** |

| ≥5 | 29 | 25 | 4 |  |  | ≥6 | 41 | 24 | 17 |  |
| --- | --- | --- | --- | --- | --- | --- | --- | --- | --- | --- |
| <5 | 84 | 57 | 27 |  |  | <6 | 72 | 58 | 14 |  |
| **DBIL**  **(μmol/mL)** |  |  |  | 0.489 | **IHC score of vimentin** |  |  |  |  | **0.049*** |
| >8 | 11 | 7 | 4 |  |  | ≥6 | 38 | 32 | 6 |  |
| ≤8 | 102 | 75 | 27 |  |  | <6 | 75 | 55 | 25 |  |
| **TNM stage**  I&II | 82 | 57 | 27 | 0.240 |  |  |  |  |  |  |
| III&IV | 31 | 25 | 6 |  |  |  |  |  |  |  |

**Supplementary Table 5. Gene ontology (GO) and pathway analysis**

| **Category** | **GOID** | **Description** | **GeneRatio** | **BgRatio** | ***p*-value** | **padj geneName** Count |
| --- | --- | --- | --- | --- | --- | --- |
| BP | GO:2000147 | positive regulation of cell motility | 44/794 | 416/14260 | 3.15E-05 | CXCL6/CEMIP/WNT5B/DEFB1/HMOX1/CARMIL2/PGF/CEACAM6/NTN1/PLPP3/CXCL8/A  0.051985226 DRA2A/ATOH8/DAPK2/SPARC/RET/NCKAP1L/BMP7/HDAC6/HDAC9/F10/RARRES2/GPE 44  R1/CORO1A/AKT1/PHPT1/CREB3/ELP6/CCBE1/COL1A1/FAM89B/ANGPT4/CXCL5/HRAS/ |

BP GO:0051272

positive regulation of cellular component movement

# 44/794 426/14260 5.57E-05 0.051985226

# GAS6/HSPB1/SPHK1/TNFSF18/SNAI1/CIB1/TRPV4/RHOD/HYAL1/MIEN1 CXCL6/CEMIP/WNT5B/DEFB1/HMOX1/CARMIL2/PGF/CEACAM6/NTN1/PLPP3/CXCL8/A DRA2A/ATOH8/DAPK2/SPARC/RET/NCKAP1L/BMP7/HDAC6/HDAC9/F10/RARRES2/GPE

# R1/CORO1A/AKT1/PHPT1/CREB3/ELP6/CCBE1/COL1A1/FAM89B/ANGPT4/CXCL5/HRAS/ 44

# GAS6/HSPB1/SPHK1/TNFSF18/SNAI1/CIB1/TRPV4/RHOD/HYAL1/MIEN1 CXCL6/CEMIP/WNT5B/DEFB1/HMOX1/CARMIL2/PGF/CEACAM6/NTN1/PLPP3/CXCL8/A DRA2A/ATOH8/DAPK2/SPARC/RET/NCKAP1L/BMP7/HDAC6/HDAC9/F10/RARRES2/GPE

BP GO:0040017 positive regulation of locomotion 44/794 441/14260 0.000124284 0.064415331

BP GO:0030335 positive regulation of cell migration 41/794 403/14260 0.000137964 0.064415331

regulation of signaling receptor

| 36/794 | 358/14260 | 0.000432171 | 0.107783614 |
| --- | --- | --- | --- |
| 37/794 | 406/14260 | 0.002189586 | 0.212326849 |

BP GO:0010469

activity

BP GO:0044057 regulation of system process

regulation of anatomical structure

[R1/CORO1A/AKT1/PHPT1/CREB3/ELP6/CCBE1/COL1A1/FAM89B/ANGPT4/CXCL5/HRAS/ 44](#_TOC_250000)

GAS6/HSPB1/SPHK1/TNFSF18/SNAI1/CIB1/TRPV4/RHOD/HYAL1/MIEN1 CXCL6/CEMIP/WNT5B/HMOX1/CARMIL2/PGF/CEACAM6/PLPP3/CXCL8/ADRA2A/ATOH 8/DAPK2/SPARC/RET/NCKAP1L/BMP7/HDAC6/HDAC9/F10/RARRES2/GPER1/CORO1A/A

KT1/CREB3/ELP6/CCBE1/COL1A1/FAM89B/ANGPT4/CXCL5/HRAS/GAS6/HSPB1/SPHK1/T 41

NFSF18/SNAI1/CIB1/TRPV4/RHOD/HYAL1/MIEN1 CXCL6/ADRA2C/ARC/PGF/CXCL8/BMP6/CTGF/ADRA2A/NDP/ERFE/FGF9/CCL2/BMP7/U CN2/HDAC6/CERS1/STC1/CCL28/AREG/MIF/JAG2/ZFYVE28/MAPK8IP2/F2/CACNG4/CXC 36

L5/GFER/GAS6/IL1RN/NEURL1/TNFSF18/ADRB2/ADM/NBL1/IL11/PMCHL2 IGFBP5/ADRA2C/BDKRB2/KCNIP3/BMP6/KISS1/CTGF/ADRA2A/ANXA6/CAMK2B/C12orf

57/STC1/NR1H3/GPER1/TBX2/SNTA1/DES/AKT1/ATP2A3/CACNA1H/RAB11FIP1/PIN1/SLC 37

9A3R1/CACNG4/GAS6/SPHK1/RAB11FIP5/FXYD2/FOXN4/OPRL1/CYBA/ADRB2/TNFRSF2

1/RANGRF/CALM3/ADM/AQP1 SCIN/ADRA2C/HMOX1/CARMIL2/NTN1/BDKRB2/ADRA2A/TRPV2/PLXNA4/PRR16/RET/L AMTOR4/NCKAP1L/GPER1/CORO1A/USH1C/AKT1/RAC3/SERPINF2/OLFM1/GPX1/SLC9A

BP GO:0090066

BP GO:1901615

size

organic hydroxy compound metabolic process

37/794 432/14260 0.006093665 0.297761278

37/794 433/14260 0.006319495 0.297761278

# 3R1/PEX11G/TMSB10/SLC12A7/RILP/TWF2/ADRB2/CDC42EP4/TRPV4/ADM/PDXP/AQP1/ 37

# BAIAP2L2/SPTB/PICK1/RTN4R APOL1/AKR1B10/BMP6/PTH1R/LIPC/PCK1/ABCG1/DGAT2/LDHB/SULT1A2/AKR1C1/GPE

# R1/IMPA2/MRS2/MAOA/ACAA1/CACNA1H/PDXK/GALK1/PNKD/DISP3/PMVK/CYP3A4/A 37

# POL2/BAAT/SNCB/BCO1/SPHK1/SLC27A5/CYP7B1/ALDH1A1/SNAI1/CEBPA/MSMO1/ITP

# KA/PDXP/NPC1 ADCY5/ADRA2C/DDO/SSTR5/BMP6/DHRS2/KISS1/ADRA2A/PON3/CLTRN/GLUL/ANO1/D GAT2/BRSK2/AKR1C1/GPER1/ACAA1/CACNA1H/PHPT1/RAB11FIP1/BAD/DHRS11/STUB1

BP GO:0010817 regulation of hormone levels 36/794 423/14260 0.007452226 0.316313109

BP GO:0009636 response to toxic substance

| 35/794 | 415/14260 | 0.009429598 | 0.331028536 |
| --- | --- | --- | --- |
| 35/794 | 416/14260 | 0.009766433 | 0.337773884 |

negative regulation of

BP GO:0042326

phosphorylation

# /HNF4A/RAPGEF4/TSPO/CYP3A4/AKR1B15/IL1RN/BCO1/RAB11FIP5/ALDH1A1/TRPV4/A 36

# RL2/ADM/IL11 HMOX1/DHRS2/APOM/GPX2/PON3/NUPR1/MT2A/SPARC/PTGS1/BMP7/HDAC6/LTC4S/CE RS1/SLC23A1/HDAC9/AREG/INMT/ACAA1/FIS1/MT1E/NXN/BAD/CLDN1/COL1A1/GPX1/S 35

# DC1/MPST/PRDX5/GPX4/PRDX2/KLF2/ENDOG/AQP1/SNN/SELENOW

# BDKRB2/PLPP3/PKN1/C1QL4/PARD6A/BMP7/PRR5L/RGS14/GPS2/SFN/GPER1/AHSG/ZFY VE28/AKT1/TP73/PIN1/CDKN2D/SLC9A3R1/GCKR/DUSP19/HSPB1/CDKN2C/TRIB1/MVP/ 35

# ARRB2/CEBPA/CIB1/FKBP8/SOCS1/CALM3/DUSP10/HYAL2/TRIB3/SIRT2/RTN4R

BP GO:0009306 protein secretion 36/794 444/14260 0.015156248 0.387583891

BP GO:0006935 chemotaxis 38/794 477/14260 0.016602866 0.395748934

BP GO:0042330 taxis 38/794 478/14260 0.017109218 0.396878696

BP GO:0002790 peptide secretion 37/794 465/14260 0.018226122 0.40330693

# ADCY5/ADRA2C/SSTR5/RAB37/BMP6/ADRA2A/RAB26/CLTRN/GLUL/IL1R2/ANO1/NR1H

# 3/BRSK2/MIF/GPER1/GSDMD/MAPK11/PHPT1/RAB11FIP1/BAD/HNF4A/RAPGEF4/RAB15/ 36 IRF3/GAS6/IL1RN/RAB11FIP5/NECAB3/IDH2/IL17RC/TNFRSF21/TRPV4/SOCS1/ARL2/NO

# X5/HYAL2 CXCL6/EPHB1/RAP1GAP/DEFB1/PGF/NTN1/CXCL8/MPP1/EPHB3/PLXNA4/DAPK2/GFRA2

# /RET/TUBB3/NCKAP1L/CCL2/BMP7/CCL28/MIF/RARRES2/CORO1A/RAC3/CREB3/ITGB2/ 38

# ARHGEF16/CXCL5/LYPLA2/HRAS/GAS6/HSPB1/SEMA6C/CYP7B1/IL17RC/ARRB2/TRPV4

# /NBL1/DOK1/SPTB CXCL6/EPHB1/RAP1GAP/DEFB1/PGF/NTN1/CXCL8/MPP1/EPHB3/PLXNA4/DAPK2/GFRA2

# /RET/TUBB3/NCKAP1L/CCL2/BMP7/CCL28/MIF/RARRES2/CORO1A/RAC3/CREB3/ITGB2/ 38

# ARHGEF16/CXCL5/LYPLA2/HRAS/GAS6/HSPB1/SEMA6C/CYP7B1/IL17RC/ARRB2/TRPV4

# /NBL1/DOK1/SPTB ADCY5/ADRA2C/SSTR5/RAB37/BMP6/KISS1/ADRA2A/RAB26/CLTRN/GLUL/IL1R2/ANO1

# /NR1H3/BRSK2/MIF/GPER1/GSDMD/MAPK11/PHPT1/RAB11FIP1/BAD/HNF4A/RAPGEF4/R 37

# AB15/IRF3/GAS6/IL1RN/RAB11FIP5/NECAB3/IDH2/IL17RC/TNFRSF21/TRPV4/SOCS1/ARL

# 2/NOX5/HYAL2

**Supplementary Table 6. Reactome pathway analysis**

| **Reactome ID** | **Description** | **Gene Ratio** | **Bg Ratio** | ***p*-value** | **padj** |
| --- | --- | --- | --- | --- | --- |
| R-HSA-2142753 | Arachidonic acid metabolism | 9/522 | 48/8809 | 0.001767732 | 0.424644378 |
| R-HSA-1980143 | Signaling by NOTCH1 | 11/522 | 72/8809 | 0.003189837 | 0.424644378 |
| R-HSA-909733 | Interferon alpha/beta signaling | 9/522 | 53/8809 | 0.003590769 | 0.424644378 |
| R-HSA-6785807 | Interleukin-4 and 13 signaling | 12/522 | 87/8809 | 0.004999739 | 0.424644378 |
| R-HSA-2644602 | Signaling by NOTCH1 PEST Domain Mutants in Cancer | 9/522 | 57/8809 | 0.005910453 | 0.424644378 |
| R-HSA-2644603 | Signaling by NOTCH1 in Cancer | 9/522 | 57/8809 | 0.005910453 | 0.424644378 |

**Gene Name KEGG ID Count**

R-HSA-2644606 Constitutive Signaling by NOTCH1

PEST Domain Mutants

R-HSA-2894858 Signaling by NOTCH1 HD+PEST

Domain Mutants in Cancer

R-HSA-2894862 Constitutive Signaling by NOTCH1

HD+PEST Domain Mutants

# PLA2G4A/ALOX5/GPX2/PON3/PTGS1/GGT1/LTC4S/GPX1/

# CEACAM6/SELPLG/ESAM/MIF/SLC16A3/F2/ITGB2/COL1A 1/SDC1/ANGPT4/HRAS/GAS6/PICK1/PROCR

| GPX4 | 876/2879 |
| --- | --- |
| DLL4/HDAC6/PSENEN/HDAC9/MAMLD1/JAG2/NEURL1B/ | 54567/10013/55851/9734/10046/3714/5 |
| TLE4/NEURL1/ARRB2/HDAC11 | 4492/7091/9148/409/79885 |

| IFI6/IFITM3/ISG15/IFI35/IFITM2/IRF3/ISG20/PSMB8/SOCS1 | 2537/10410/9636/3430/10581/3661/366  9/5696/8651 |
| --- | --- |
| F13A1/HMOX1/CXCL8/ALOX5/MMP2/CCL2/MAOA/AKT1/I | 2162/3162/3576/240/4313/6347/4128/2 |
| TGB2/BIRC5/RORC/SOCS1 | 07/3689/332/6097/8651 |
| DLL4/HDAC6/PSENEN/HDAC9/MAMLD1/JAG2/NEURL1B/ | 54567/10013/55851/9734/10046/3714/5 |
| NEURL1/HDAC11 | 4492/9148/79885 |
| DLL4/HDAC6/PSENEN/HDAC9/MAMLD1/JAG2/NEURL1B/ | 54567/10013/55851/9734/10046/3714/5 |
| NEURL1/HDAC11 | 4492/9148/79885 |
| DLL4/HDAC6/PSENEN/HDAC9/MAMLD1/JAG2/NEURL1B/ | 54567/10013/55851/9734/10046/3714/5 |
| NEURL1/HDAC11 | 4492/9148/79885 |
| DLL4/HDAC6/PSENEN/HDAC9/MAMLD1/JAG2/NEURL1B/ | 54567/10013/55851/9734/10046/3714/5 |
| NEURL1/HDAC11 | 4492/9148/79885 |
| DLL4/HDAC6/PSENEN/HDAC9/MAMLD1/JAG2/NEURL1B/ | 54567/10013/55851/9734/10046/3714/5 |
| NEURL1/HDAC11 | 4492/9148/79885 |

| 9/522 | 57/8809 | 0.005910453 | 0.424644378 |
| --- | --- | --- | --- |
| 9/522 | 57/8809 | 0.005910453 | 0.424644378 |
| 9/522 | 57/8809 | 0.005910453 | 0.424644378 |

| R-HSA-202733 | Cell surface interactions at the | 14/522 | 112/8809 | 0.006223448 | 0.424644378 |
| --- | --- | --- | --- | --- | --- |
|  | vascular wall |  |  |  |  |
| R-HSA-114608 | Platelet degranulation | 14/522 | 114/8809 | 0.00726962 | 0.424644378 |
| R-HSA-76005 | Response to elevated platelet cytosolic Ca^2+^  Metabolism of amino acids and | 14/522 | 118/8809 | 0.009779839 | 0.424644378 |

# F13A1/ECM1/SPARC/RARRES2/AHSG/CFD/SPP2/SERPINF2

# /SERPINA3/GAS6/TEX264/CALM3/CHID1/CD63 F13A1/ECM1/SPARC/RARRES2/AHSG/CFD/SPP2/SERPINF2

# /SERPINA3/GAS6/TEX264/CALM3/CHID1/CD63

# PRODH2/CBSL/DDO/PSMB9/SDSL/GLUL/RPL39L/ALDH4A 1/AZIN2/PAOX/GAMT/INMT/SERINC2/PSME2/NNMT/NMR

5321/240/2877/5446/5742/2678/4056/2 9

11

9

12

9

9

9

9

9

4680/6404/90952/4282/9123/2147/3689

/1277/6382/51378/3265/2621/9463/105 14

44

2162/1893/6678/5919/197/1675/6694/5

345/12/2621/51368/808/66005/967 14

2162/1893/6678/5919/197/1675/6694/5

345/12/2621/51368/808/66005/967 14

# R-HSA-71291

derivatives 31/522 338/8809 0.009904242 0.424644378

# AL1/MPST/SLC25A10/ASS1/RPL36/PSMB6/PSMB8/RPS2/RP L35/RPS9/RPS29/ASL/SHMT1/TST/ETHE1/RPL8

# DLL4/LFNG/HDAC6/PSENEN/HDAC9/MAMLD1/JAG2/ATP

| 58510/102724560/8528/5698/113675/2 |  |
| --- | --- |
| 752/116832/8659/113451/196743/2593/ |  |
| 11185/347735/5721/4837/57407/4357/1 | 31 |
| 468/445/25873/5694/5696/6187/11224/ |  |
| 6203/6235/435/6470/7263/23474/6132 |  |
| 54567/3955/10013/55851/9734/10046/3 |  |
| 714/489/54492/7091/9148/1869/409/79 | 14 |

R-HSA-157118 Signaling by NOTCH 14/522 120/8809 0.011267357 0.45356459

| 2A3/NEURL1B/TLE4/NEURL1/E2F1/ARRB2/HDAC11 | 885 |
| --- | --- |
| F13A1/RASGRP2/ECM1/ADRA2C/PLA2G4A/ADRA2A/SPA | 2162/10235/1893/152/5321/150/6678/5 |
| RC/RARRES2/AHSG/F2/AKT1/CFD/SPP2/COL1A1/SERPINF | 919/197/2147/207/1675/6694/1277/534 23 |
| 2/SERPINA3/RAPGEF4/GAS6/TEX264/ARRB2/CALM3/CHI | 5/12/11069/2621/51368/409/808/66005/ |
| D1/CD63 | 967 |

R-HSA-76002 Platelet activation, signaling and

aggregation

23/522 234/8809 0.01146033 0.45356459
