# Supplementary material for: Low SVEP1 in intrahepatic cholangiocarcinoma mediates phenotype switching-driven metastasis by Jag2/Notch1/Hes5
Source: Cell Death Dis. 2025 Nov 28;16(1):871. doi: 10.1038/s41419-025-08170-2 (PMC12663138; doi:10.1038/s41419-025-08170-2)
Supplement: Supplementary file 3 — Supplementary figures [file 41419_2025_8170_MOESM3_ESM.docx]

**Supplementary figures**

**
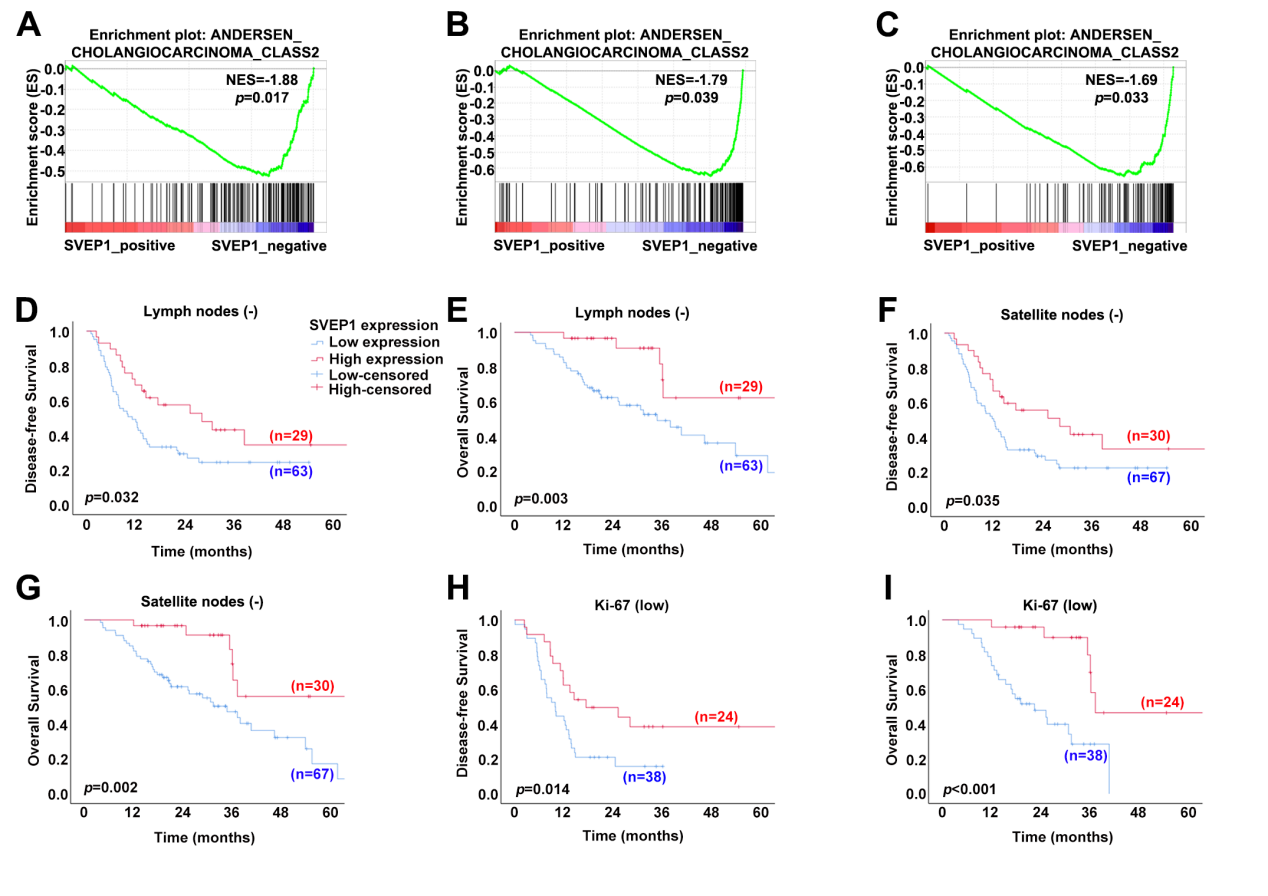
**

**Supplementary Fig. 1. Decreased expression of SVEP1 in CCA suggested poorer DFS and OS in overall patients and low-risk subgroups (negative lymph nodes and satellite nodes and low Ki-67 expression).**

(A–C). GSEA from datasets including GSE132305 (A), GSE89749 (B), and GSE45001 (C) indicates that reduced expression of SVEP1 is negatively correlated with the clinical prognosis of CCA. (D and E). Kaplan–Meier survival curves illustrate the correlation between SVEP1 expression and 5-year DFS (D) and OS (E) in CCA patients with negative lymph nodes metastasis. (F and G). Kaplan–Meier survival curves illustrate the correlation between SVEP1 expression and 5-year DFS (F) and OS (G) in CCA patients with negative satellite nodes. (H and I). Kaplan–Meier survival curves illustrate the correlation between SVEP1 expression and 5-year DFS (H) and OS (I) in CCA patients with low Ki-67 expression.


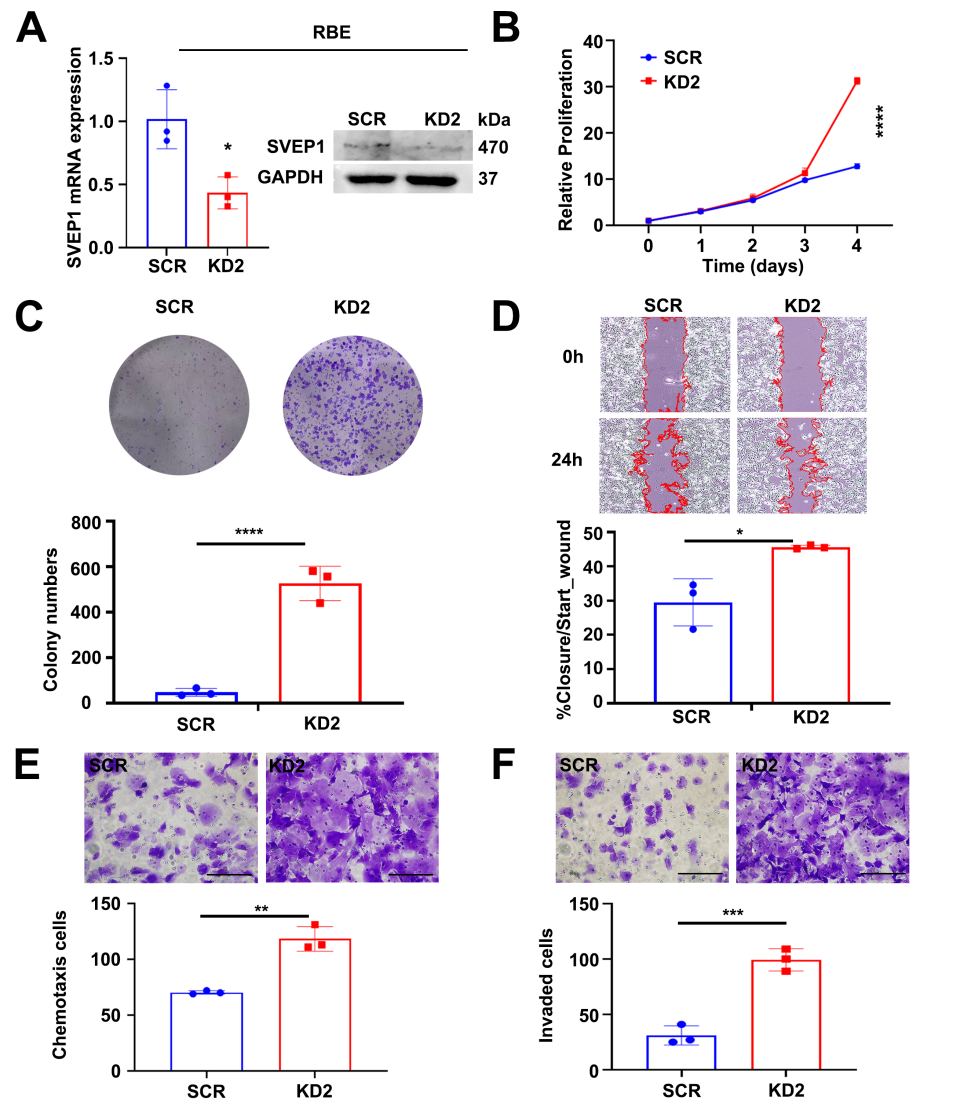


**Supplementary Fig. 2. Down-regulation of SVEP1 in RBE cells increases cell proliferation, migration, and invasion in vitro.**

(A). WB and RT-PCR analyses showing the SVEP1 knocked down clone2 obtained in RBE cell line, denoted as KD2 (short for SVEP1 KD2). GAPDH was used as a loading control. (B). CCK-8 assay exhibiting differences in proliferation ability between SCR and KD2 groups in RBE cell line. (C). Representative images of 2D colony formation assays in RBE cells transfected with SVEP1 or control shRNAs. (D). Representative images and quantification of wound closure in RBE (time = 24 h) cells transfected with SVEP1 or control shRNAs after scratching. (E). Representative images of chemotactic migration (transwell assay) in RBE cells transfected with SVEP1 or control shRNAs (Scale bars, 100μm). (F). Cell invasion assay results comparing SCR and KD2 groups in RBE cells using transwell filter chambers. Representative images show invaded cells were calculated (Scale bars, 100 μm). **p* < 0.05, ***p* < 0.01, ****p* < 0.001, *****p* < 0.0001.

**
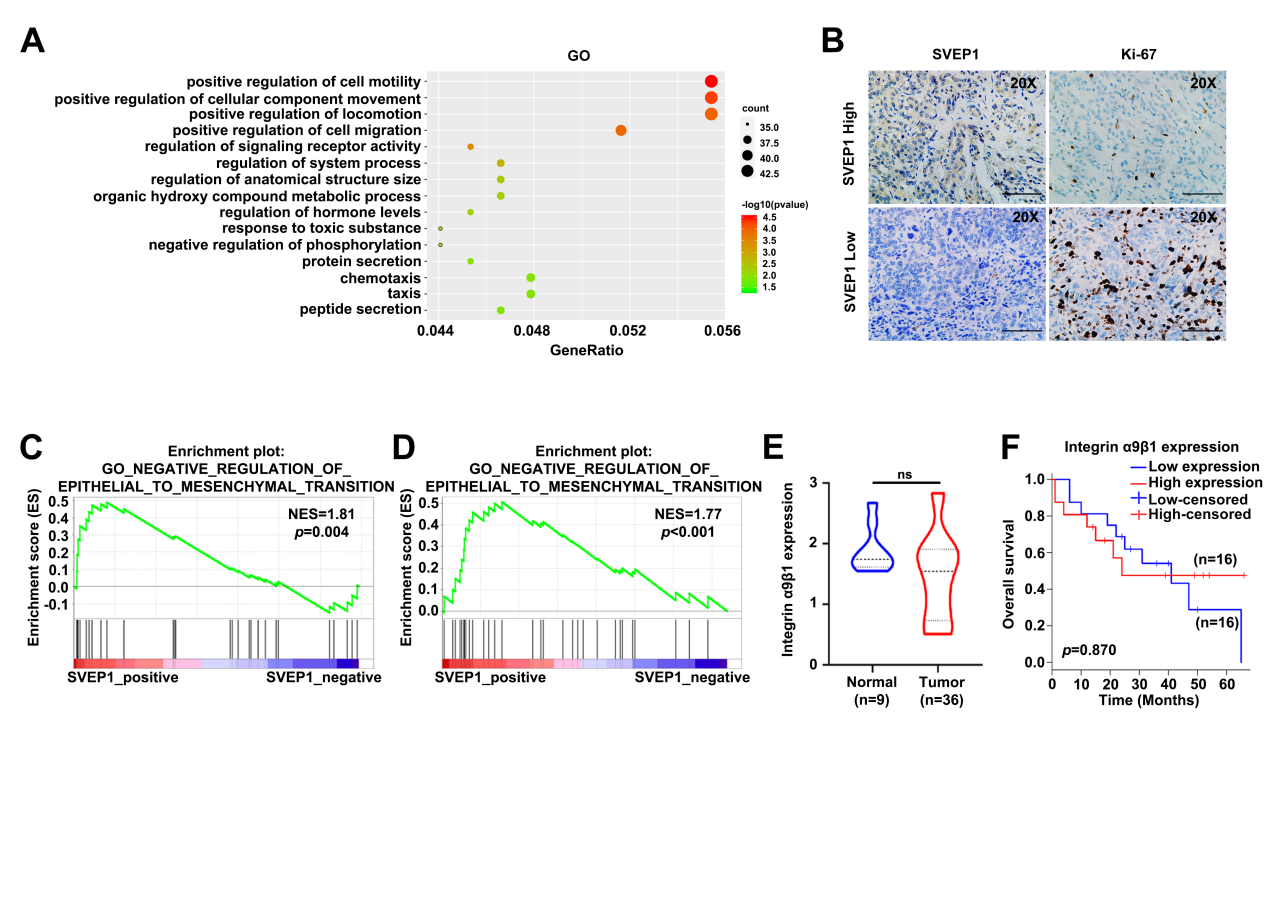
**

**Supplementary Fig. 3. Correlation between SVEP1 expression, biological characteristics of ICC, and identification of its receptor, integrin α9β1, expression in ICC.**

(A). GO pathways enriched in genes significantly associated with SVEP1 depletion in RBE cells. Bubble colors represent the adjusted *p*-value (red = most significant). The rich factor reflects the proportion of enriched genes for each term. (B). Representative images display varying levels of SVEP1 and Ki-67 expression in 113 ICC samples (Scale bars, 100 μm). (C and D). Analysis of GSE107102 (C) and GSE89749 (D) datasets showing that SVEP1 expression is negatively correlated with genes associated with EMT. (E). Analysis of integrin α9β1 mRNA levels in normal and tumor tissues in ICC using TCGA database. (F). Kaplan–Meier survival analysis illustrating the correlation between integrin α9β1 expression and 5-year OS in ICC using TCGA datasets.
