# Supplementary figures and images for: Low SVEP1 in intrahepatic cholangiocarcinoma mediates phenotype switching-driven metastasis by Jag2/Notch1/Hes5
Source: Cell Death Dis. 2025 Nov 28;16(1):871. doi: 10.1038/s41419-025-08170-2 (PMC12663138; doi:10.1038/s41419-025-08170-2)

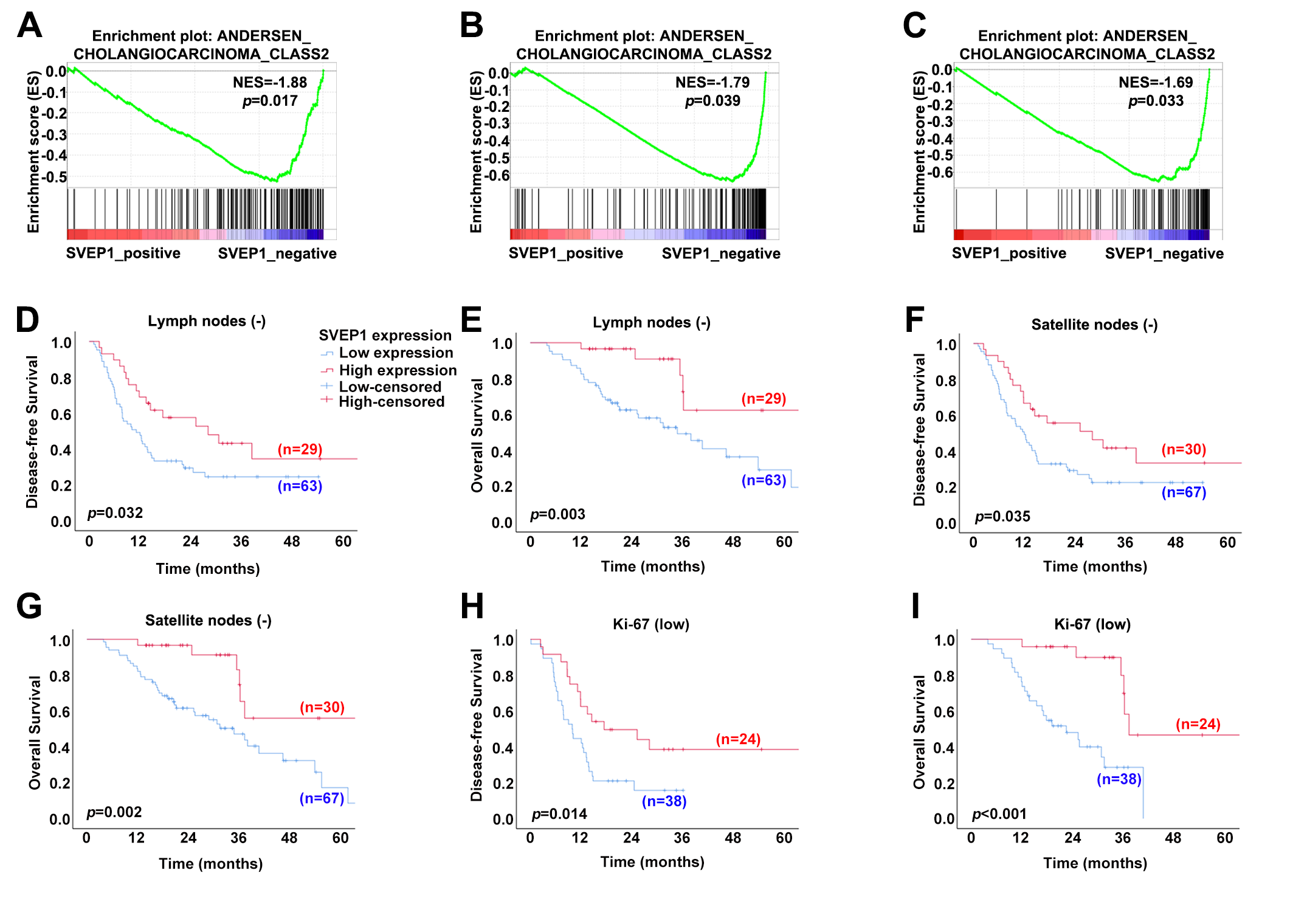

Supplement: Supplementary file 4 — Figure S1 [file 41419_2025_8170_MOESM4_ESM.tif]
