# Supplementary material for: Low SVEP1 in intrahepatic cholangiocarcinoma mediates phenotype switching-driven metastasis by Jag2/Notch1/Hes5
Source: Cell Death Dis. 2025 Nov 28;16(1):871. doi: 10.1038/s41419-025-08170-2 (PMC12663138; doi:10.1038/s41419-025-08170-2)

FIG2

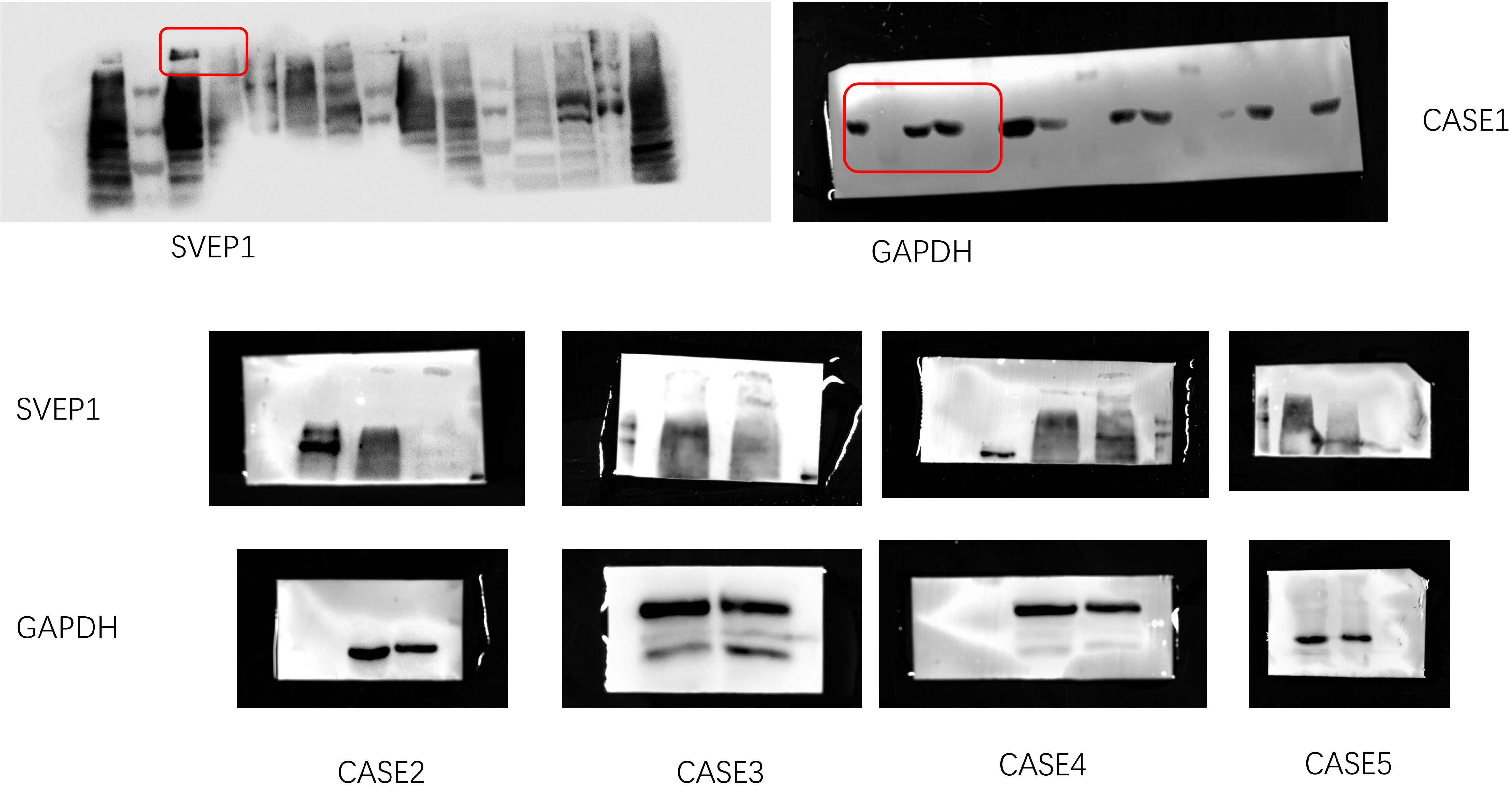

FIG3

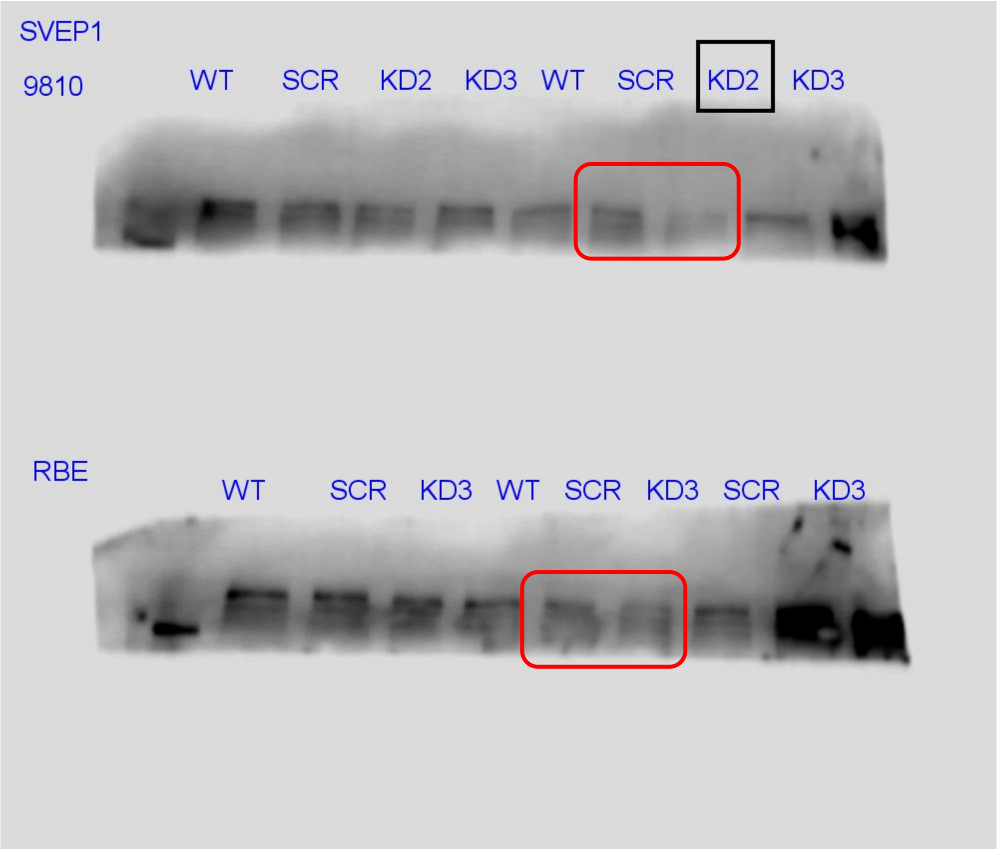
SVEP1
9810
WT SCR KD2 KD3 WT SCR KD2 KD3
RBE
WT SCR KD3 WT SCR KD3 SCR KD3

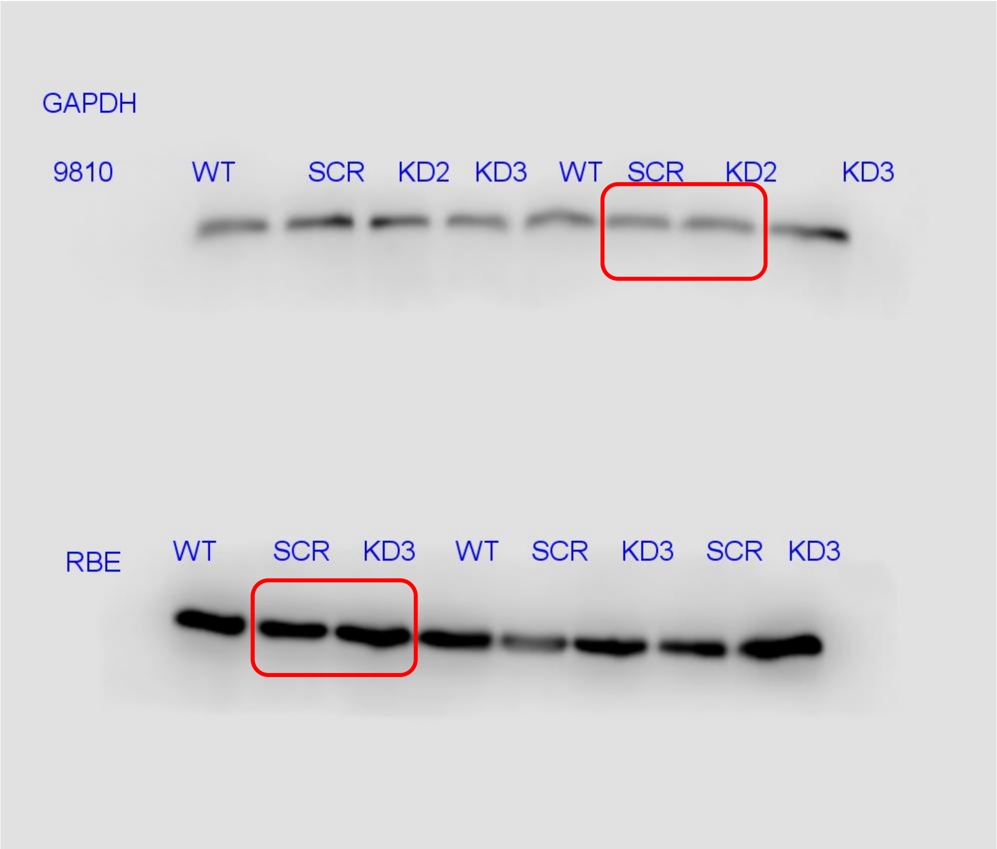
GAPDH
9810
WT SCR KD2 KD3 WT SCR KD2 KD3
RBE
WT SCR KD3 WT SCR KD3 SCR KD3

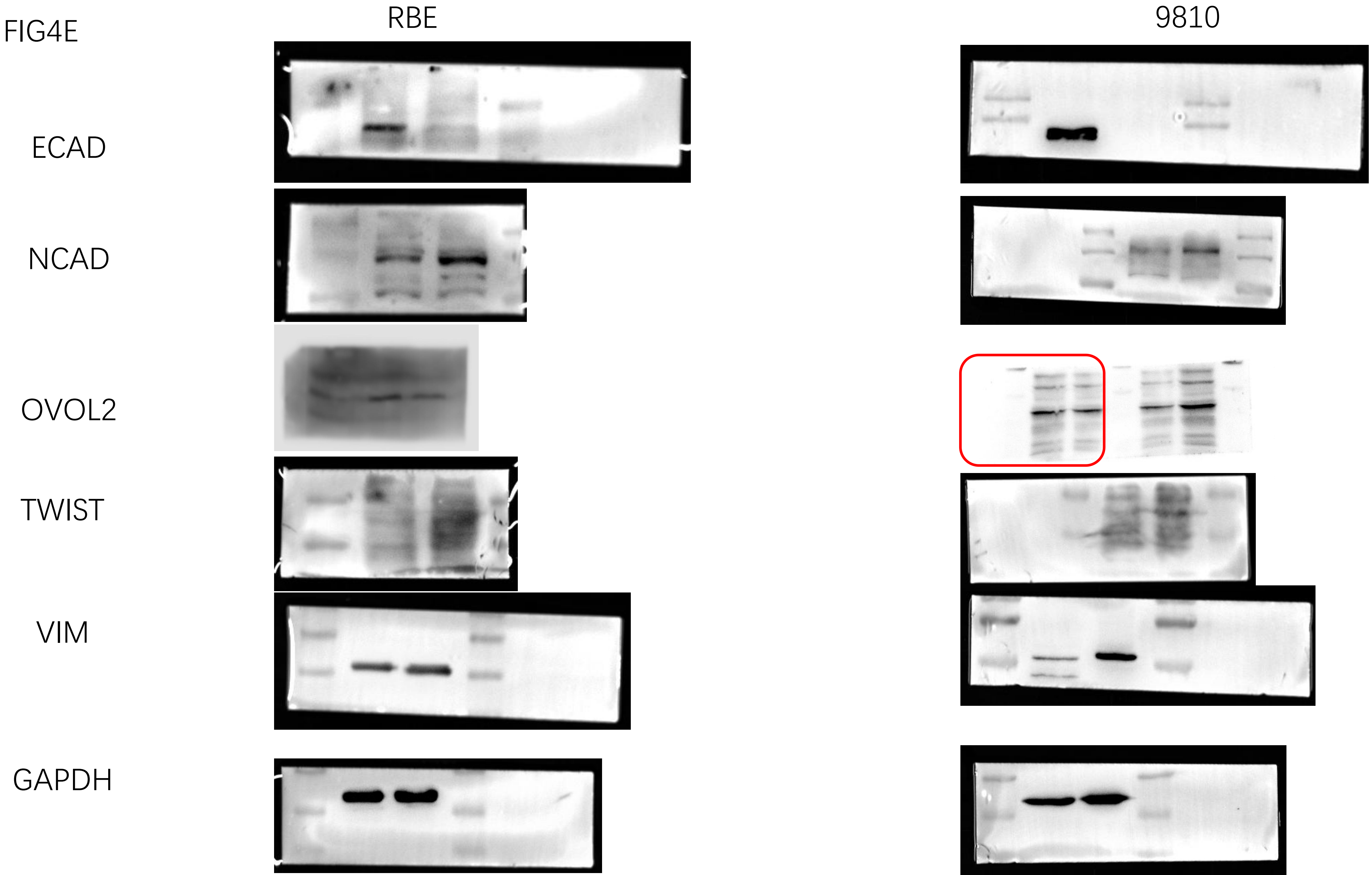
FIG4E
RBE
9810
ECAD
NCAD
OVOL2
TWIST
VIM
GAPDH

FIG6D

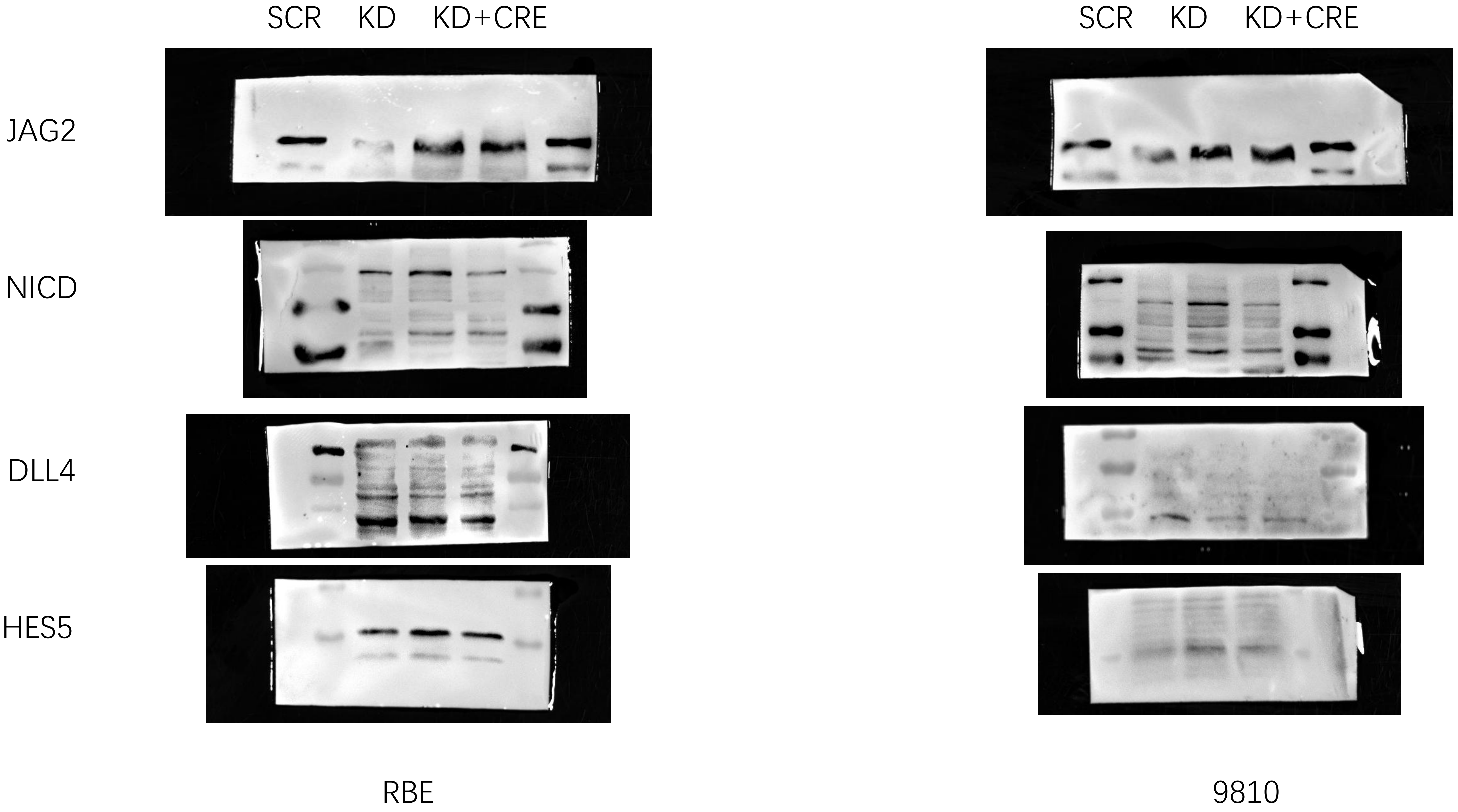

FIG6D

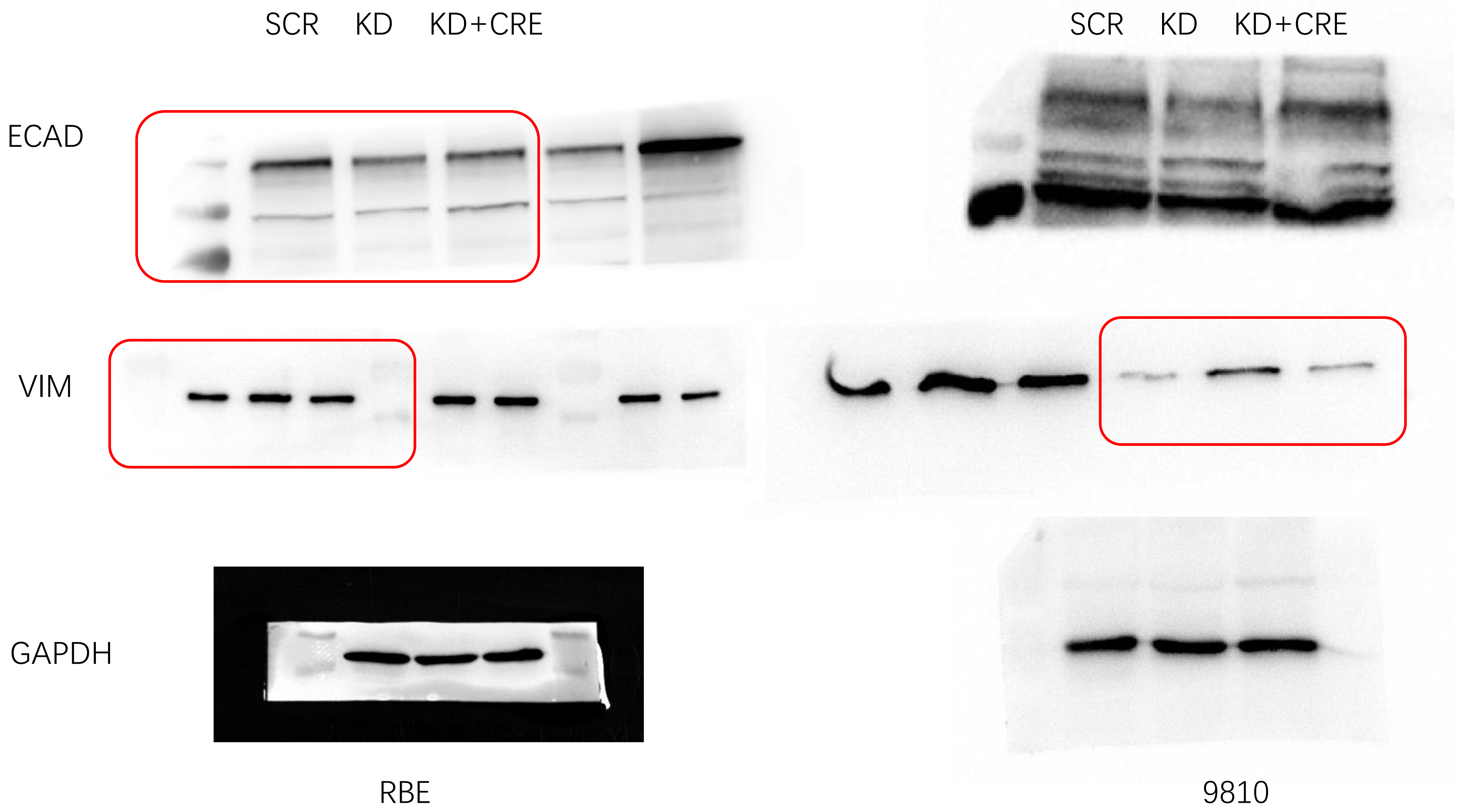

FIG7A

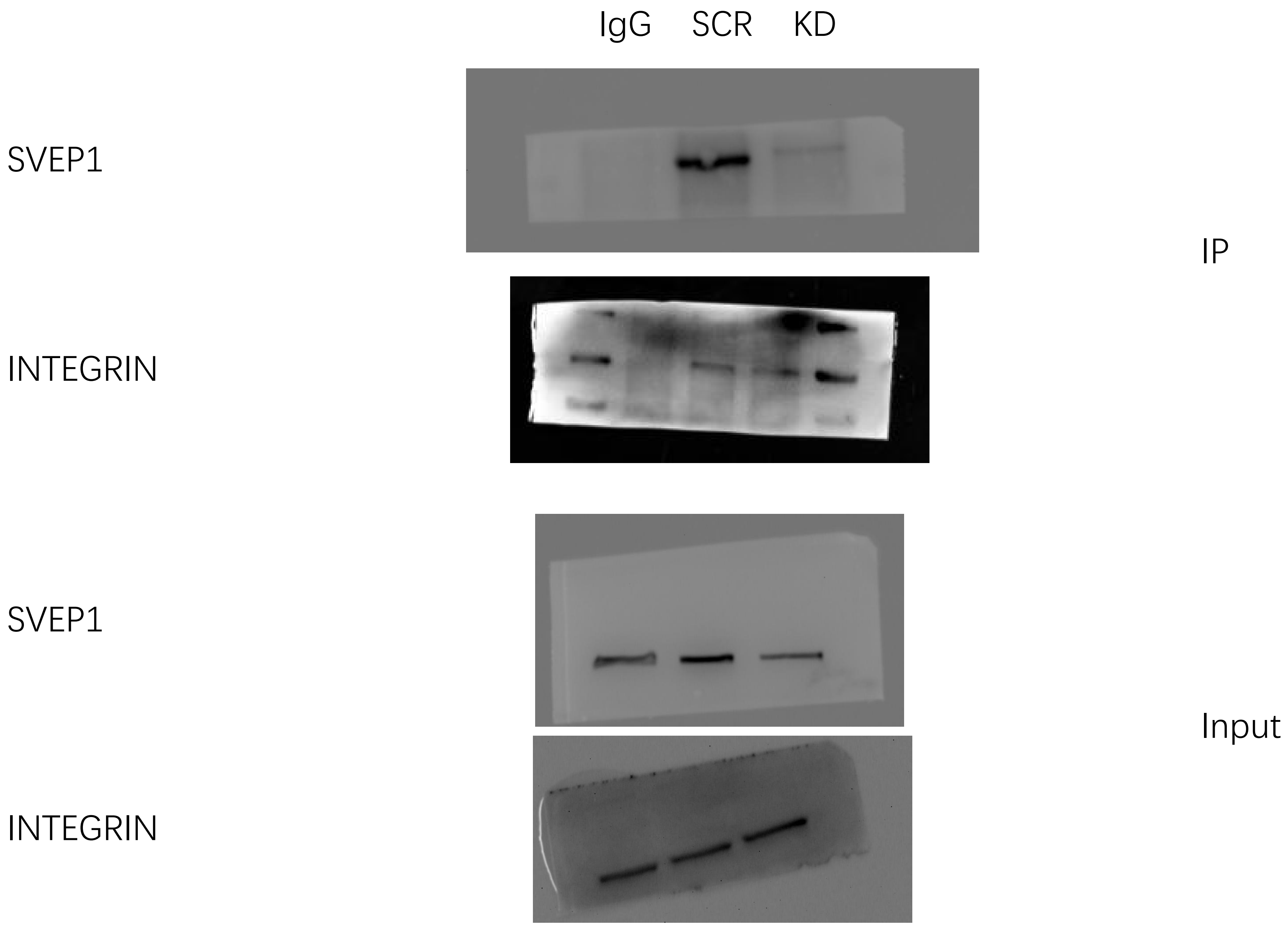

FIG7B

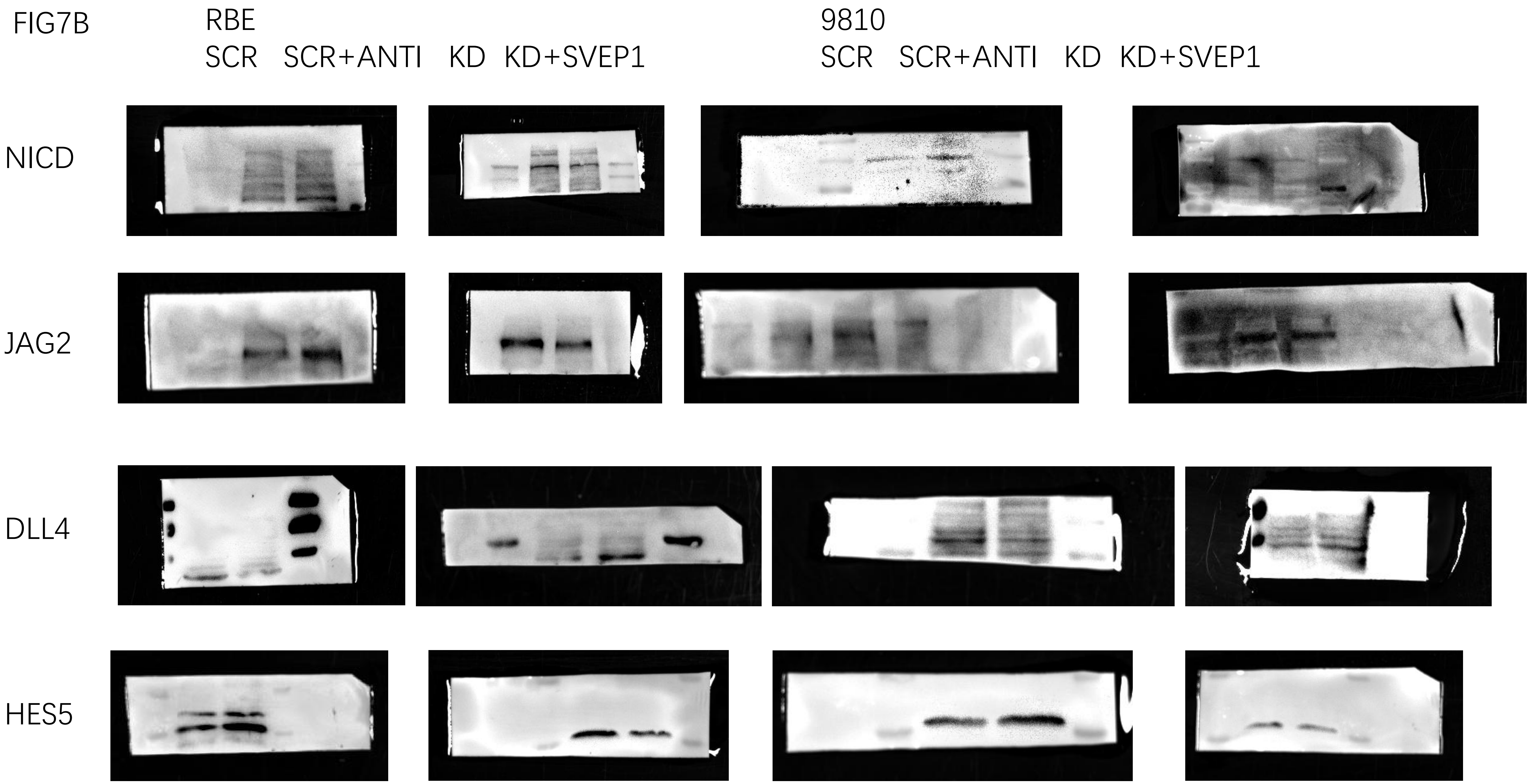
RBE
SCR SCR+ANTI KD KD+SVEP1
9810
SCR SCR+ANTI KD KD+SVEP1
NICD
JAG2
DLL4
HES5

FIG7B

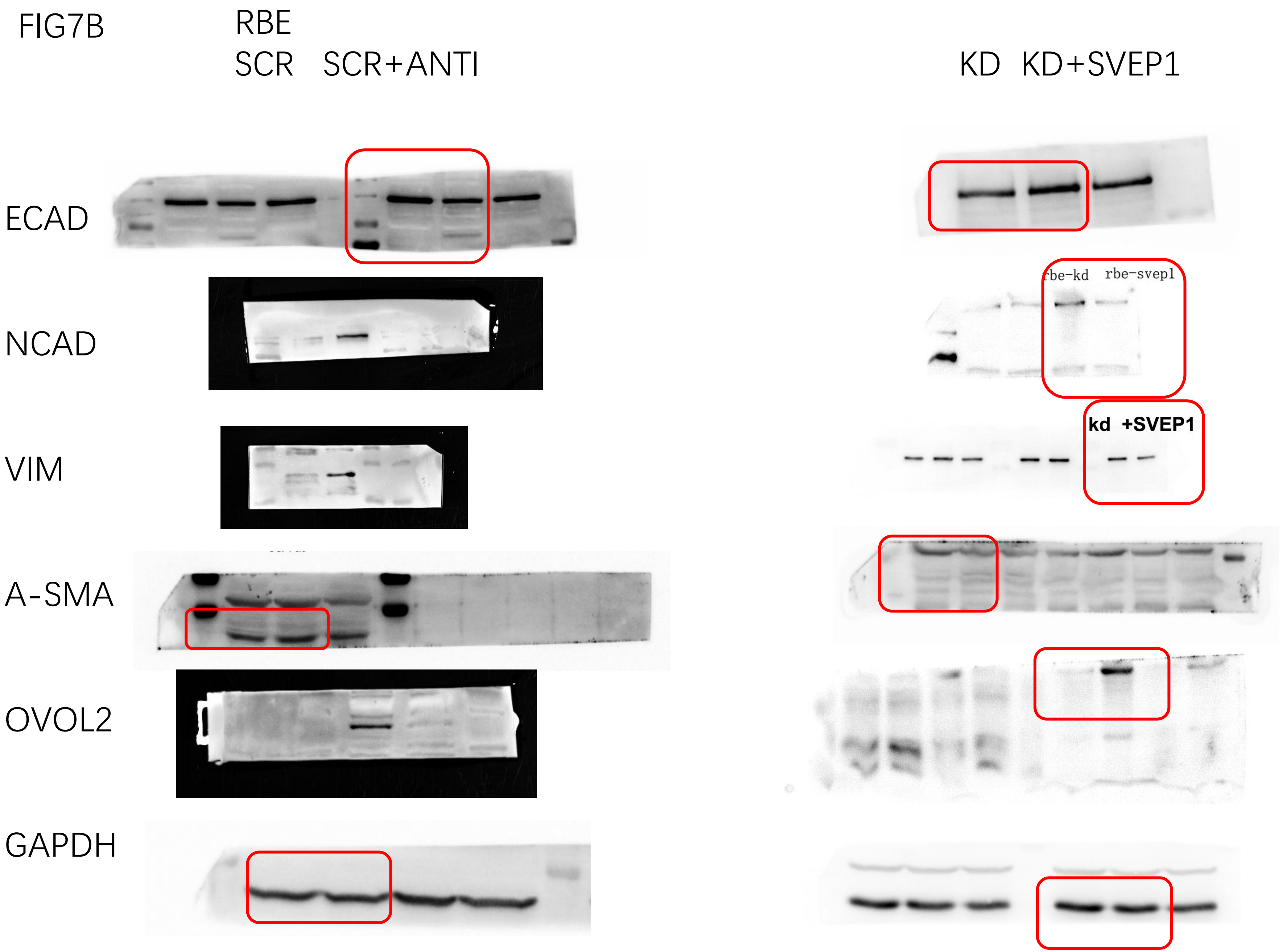
RBE
SCR
SCR+ANTI
KD
KD+SVEP1
ECAD
NCAD
VIM
A-SMA
OVOL2
GAPDH
rbe-kd
rbe-svep1
kd
+SVEP1

FIG7B

9810

SCR SCR+ANTI

KD KD+SVEP1

ECAD

NCAD

VIM

A-SMA

OVOL2

GAPDH

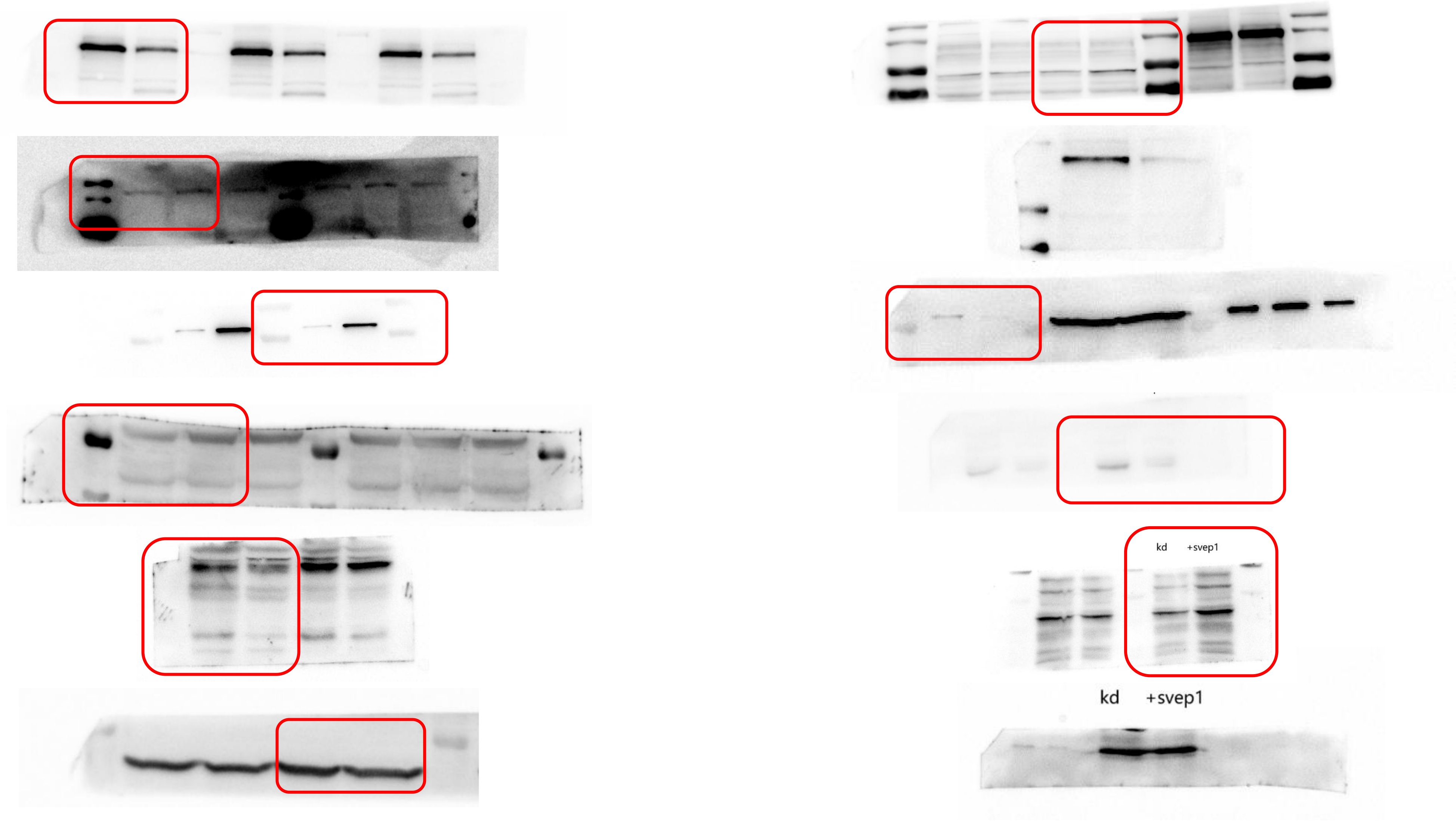

Supplement: Supplementary file 7 — WB-RAWDATA [file 41419_2025_8170_MOESM7_ESM.pdf]
